# Cat eye syndrome chromosome region, candidate 1 is a novel prognostic biomarker in hypertrophic cardiomyopathy

**DOI:** 10.1186/s43556-026-00648-5

**Published:** 2026-10-30

**Authors:** Yu Zhang, Fangfang Wan, Chen Li, Bo Wang, Mo Zhang, Minjie Lu, Lianming Kang, Liwen Liu, Jizheng Wang, Lei Song

**Affiliations:** 1 Cardiomyopathy Ward, Fuwai Hospital, Chinese Academy of Medical Sciences and Peking Union Medical College, Beijing, China; 2 State Key Laboratory of Cardiovascular Disease, Fuwai Hospital, National Center for Cardiovascular Diseases, Chinese Academy of Medical Sciences and Peking Union Medical College, Beijing, China; 3 Center of Coronary Artery Diseases, Beijing Anzhen Hospital, Capital Medical University, Beijing, China; 4 Department of Ultrasound, Xijing Hospital, Fourth Military Medical University, Xi’an, Shaanxi, China; 5 National Clinical Research Center of Cardiovascular Diseases, Fuwai Hospital, National Center for Cardiovascular Diseases, Chinese Academy of Medical Sciences and Peking Union Medical College, Beijing, China

**Keywords:** Hypertrophic cardiomyopathy, Transcriptome, CECR1, Sudden cardiac death

## Abstract

Hypertrophic cardiomyopathy (HCM) exhibits great prognostic heterogeneity, necessitating the identification of high-risk patients for timely treatment. This study aimed to find novel prognostic biomarkers directly from diseased cardiac tissue to improve risk stratification of HCM. We utilized a myocardial transcriptome discovery cohort (*n* = 246; median follow-up 6.8 years) alongside two independent plasma validation cohorts (Beijing, *n* = 1,238; follow-up 2.5 years; Xi’an, *n* = 402; follow-up 4.8 years). The primary and secondary endpoints were cardiovascular death and sudden cardiac death (SCD), respectively. In the discovery cohort, myocardial expression of *CECR1* (cat eye syndrome chromosome region, candidate 1, also known as *ADA2*, Adenosine Deaminase 2) demonstrated the strongest association with cardiovascular death (*P* = 9.6 × 10^–5^). Plasma CECR1 levels correlated modestly but significantly with myocardial expression. In the Beijing validation cohort, decreased plasma CECR1 predicted increased risks of cardiovascular death (adjusted hazard ratio (HR) 3.731, *P* < 0.001) and SCD (adjusted HR 7.398, *P* < 0.001), with areas under the curves (AUCs) of 0.720 and 0.798, respectively. These findings were replicated in the Xi’an validation cohort. Integrating plasma CECR1 into the HCM Risk-SCD model significantly enhanced its predictive performance, increasing the AUC from 0.723 to 0.845 (*P* = 0.031) in the Beijing validation cohort and consistently improving it from 0.600 to 0.781 (*P* = 0.018) in the Xi’an validation cohort. In conclusion, CECR1 represents a novel prognostic biomarker of HCM. Measurement of CECR1 may improve risk stratification by facilitating the identification of individuals at higher risk of cardiovascular mortality. CECR1 assessment may provide additional information for SCD risk evaluation and support clinical decision-making regarding implantable cardioverter-defibrillator therapy.

**Supplementary Information:**

The online version contains supplementary material available at https://doi.org/10.1186/s43556-026-00648-5.

## Introduction

Hypertrophic cardiomyopathy (HCM), the most common inherited myocardial disease, typically presents with unexplained left ventricular (LV) hypertrophy. HCM represents an important health burden [1], affecting at least 1 in 500 individuals in the general population [2]. Moreover, the annual incidence of cardiovascular death in HCM patients is approximately 1–2% [3]. HCM can be challenging to diagnose and manage because it is a highly heterogeneous disease in terms of clinical presentation and outcomes, ranging from no symptoms to severe heart failure (HF) and premature sudden cardiac death (SCD) [4]. Accurate risk stratification in patients with HCM is essential for identifying those at high risk of adverse events. This enables intensified clinical management, including enhanced monitoring of modifiable HF risk factors, optimization of medical therapy, and timely implementation of implantable cardioverter-defibrillator (ICD) therapy for SCD prevention.

Existing risk stratification strategies for HCM rely primarily on clinical history, imaging findings, and electrocardiographic parameters [2, 3, 5]; however, they do not incorporate molecular biomarkers underlying disease pathogenesis and progression. This gap may stem from the absence of direct resolution of HCM-affected myocardium in previous studies, which permits more precise characterization of pathological cardiac alterations. Investigating myocardial tissue from patients undergoing septal myectomy offers a powerful approach to identify novel, disease-driven signals that directly capture the core pathophysiological mechanisms of cardiac remodelling.

High-throughput sequencing enables comprehensive profiling of molecular signatures in targeted specimens, thereby facilitating the identification of the most clinically significant biomarkers [6]. Transcriptomic analysis via RNA sequencing (RNA-seq) allows in-depth assessment of gene expression profiles associated with prognosis in diseased lesions [7, 8]. However, this approach remains underused for identifying biomarkers predictive of cardiovascular mortality in HCM patients, especially regarding molecular profiling of the HCM-affected myocardium. To address this gap, we analysed RNA-seq data from septal tissues of an HCM myectomy cohort with the primary objective of identifying and validating novel myocardium-driven prognostic biomarkers to improve risk stratification in patients with HCM.

Through transcriptomic screening, cat eye syndrome chromosome region, candidate 1 (*CECR1*), also known as adenosine deaminase 2 (*ADA2*), emerged as the most compelling prognostic candidate. Given that detectable circulating signals are important for clinical application and that plasma CECR1 levels correspond to those in myocardial tissue, we evaluated the prognostic value of CECR1 across two plasma validation cohorts. The results revealed that decreased plasma CECR1 levels were associated with increased risks of cardiovascular death and SCD. The incorporation of plasma CECR1 levels into the conventional HCM Risk-SCD model significantly improved its predictive performance. Collectively, these findings establish CECR1 as an effective biomarker capable of refining risk stratification and informing clinical decision-making in patients with HCM.

## Results

### Study design

**Fig. 1:**
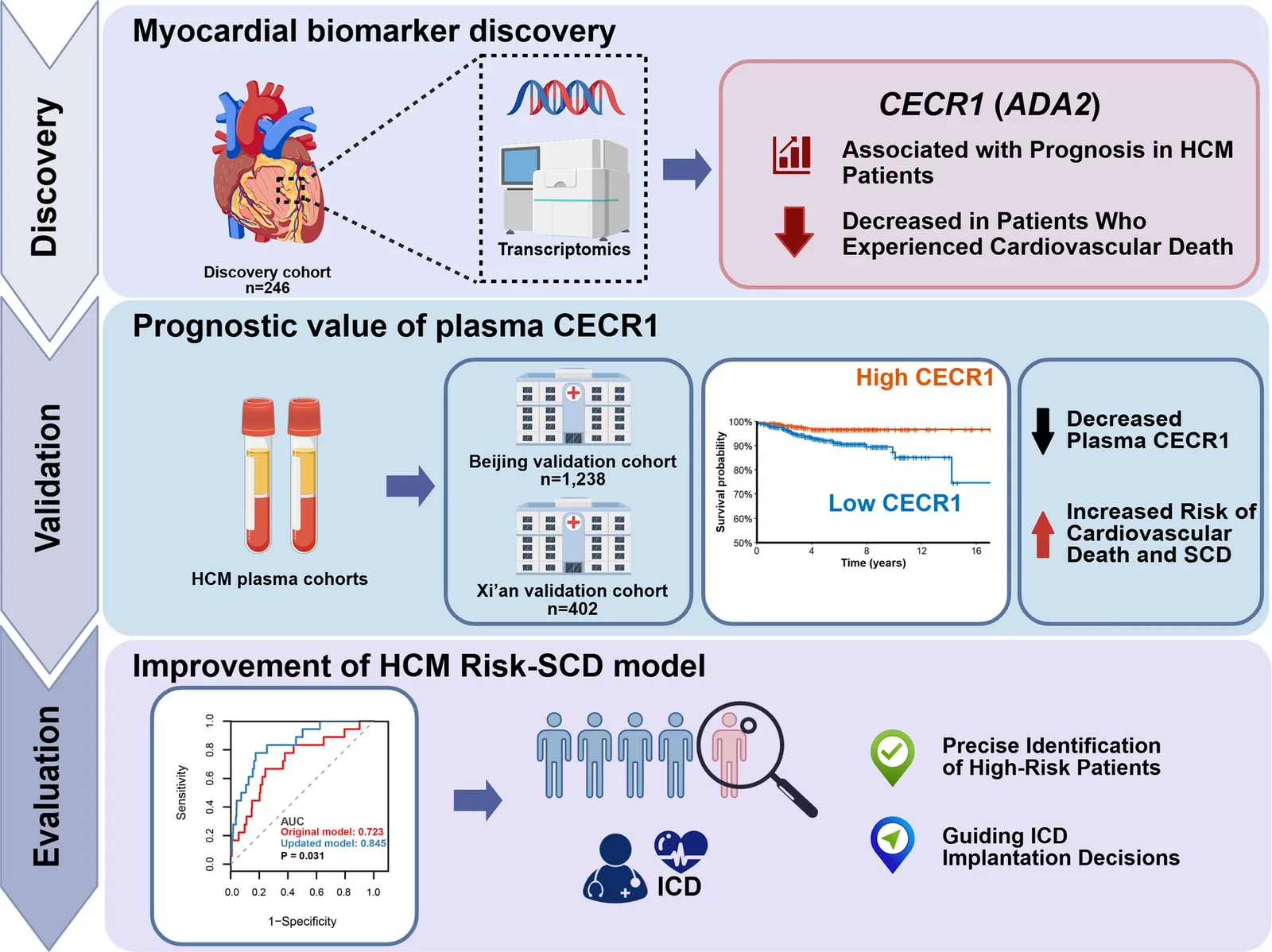
Overview of the workflow for the entire study. HCM, hypertrophic cardiomyopathy; ICD, implantable cardioverter-defibrillator; SCD, sudden cardiac death

The study was designed in three sequential phases, namely, biomarker discovery, biomarker validation, and risk evaluation (Fig. 1). The primary endpoint of this study was cardiovascular death; the secondary endpoint was SCD. First, in the biomarker discovery phase, 269 HCM patients who underwent septal myectomy at Fuwai Hospital were evaluated for myocardial transcriptomic profiling. After excluding 23 patients who were lost to follow-up, 246 patients were included in the final analysis. In this phase, we identified *CECR1* as the candidate biomarker most strongly associated with cardiovascular death. We confirmed a significant correlation between plasma CECR1 levels and its expression in myocardial tissue. In the biomarker validation phase, the prognostic value of plasma CECR1 was evaluated in two independent plasma cohorts. For the internal Beijing validation cohort, 1,339 HCM patients with plasma samples collected at enrolment were recruited from Fuwai Hospital, of whom 1,238 were included after excluding 101 patients lost to follow-up. For the external Xi’an validation cohort, 413 HCM patients with plasma samples collected at enrolment were recruited from Xijing Hospital, with 402 included in the final analysis after excluding 11 patients lost to follow-up. Finally, given the established association between plasma CECR1 and SCD in the validation phase, we evaluated whether incorporating plasma CECR1 could improve the predictive performance of the conventional HCM Risk-SCD model. Detailed inclusion and exclusion criteria for each phase are provided in the Methods and Fig. S1.

### Myocardial *CECR1* expression is associated with prognosis in patients with HCM

**Fig. 2:**
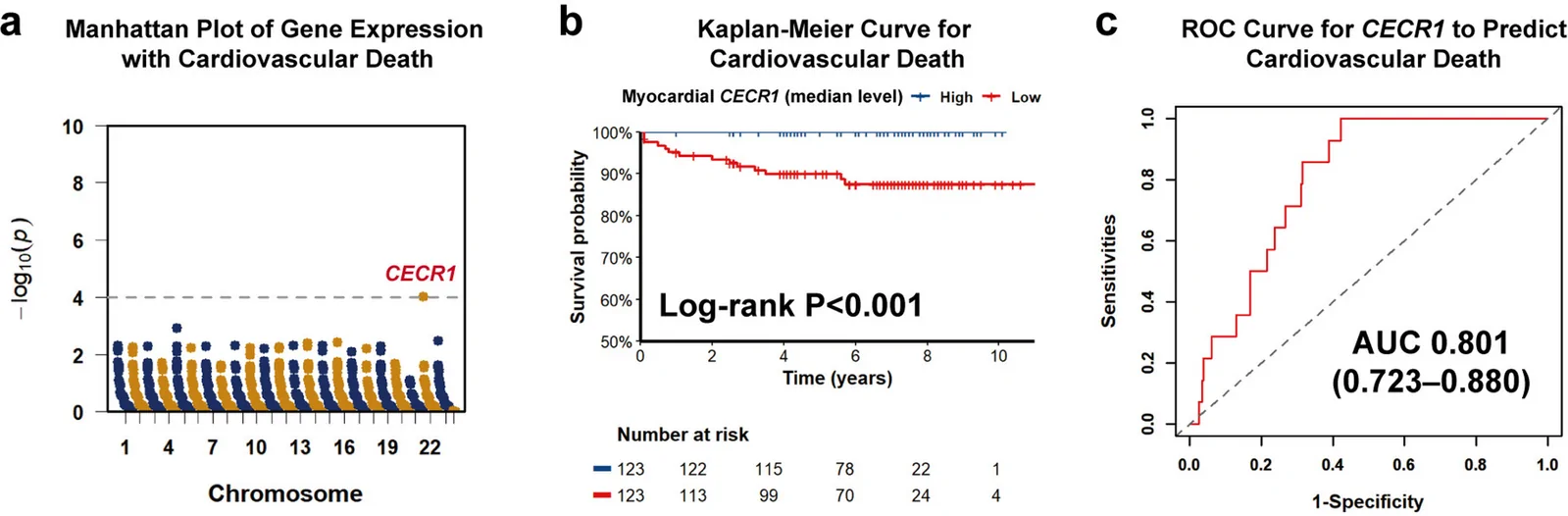
Identification of *CECR1* as a novel prognostic biomarker of HCM. **a** Manhattan plot showing that among all the genes, *CECR1* had the strongest association with cardiovascular death. **b** Kaplan‒Meier curve for cardiovascular death according to the median level of myocardial *CECR1* expression. **c** ROC curve for the ability of *CECR1* to predict cardiovascular death. These analyses included 246 patients with 14 events. AUC, area under the curve; HCM, hypertrophic cardiomyopathy; ROC, receiver operating characteristic

To identify myocardium-derived prognostic biomarkers in HCM, we performed transcriptome-wide survival screening in 246 patients from the discovery cohort with available myocardial RNA-seq and follow-up data. The baseline characteristics of the discovery cohort are presented in Table S1. During the median follow-up period of 6.8 years, 14 (5.7%) patients experienced cardiovascular death. Transcriptome-wide survival analysis based on the myocardial RNA-seq data identified *CECR1* as the gene most strongly associated with cardiovascular death among the analysed genes (Log-rank P = 9.6 × 10^–5^; Fig. 2a), although it did not achieve genome-wide significance. This finding suggests that *CECR1* may represent a candidate molecular marker associated with adverse outcomes in HCM. The full list of genes associated with cardiovascular death (*P* < 0.05) is provided in Table S2.

Patients with lower myocardial *CECR1* expression had a significantly greater incidence of cardiovascular death, as shown by the Kaplan‒Meier survival curve in Fig. 2b. Receiver operating characteristic (ROC) analysis revealed an area under the curve (AUC) of 0.801 (95% confidence interval (CI) 0.723–0.880) for the ability of *CECR1* to predict cardiovascular death (Fig. 2c). Furthermore, in the entire discovery cohort (*n* = 246), Cox regression analysis demonstrated that *CECR1* was independently negatively associated with the risk of cardiovascular death in HCM patients after adjustment (per 1 FPKM decrease: adjusted hazard ratio (HR) 1.736, 95% CI 1.275–2.364, *P* < 0.001; Table 1). To evaluate model robustness, sensitivity analyses were performed after excluding procedure-related deaths. Myocardial CECR1 expression remained independently associated with cardiovascular death (Table S3), with lower CECR1 expression associated with worse survival (Log-rank *P* < 0.001; Fig. S2a) and an AUC of 0.804 (95% CI 0.714–0.888; Fig. S2b).

**Table 1:** Associations of CECR1 with endpoints in HCM

		**Entire cohort analysis**			**Complete-case sensitivity analysis**		
**Cohort**	**Endpoint**	**Number of patients**	^*^Adjusted HR (95% CI)	^*^Adjusted *P*-value	**Number of patients**	^**†**^**Adjusted HR (95% CI)**	^**†**^**Adjusted *P*-value**
**Biomarker discovery cohort (myocardial tissue)**							
*CECR1* (per 1 FPKM decrease)	Cardiovascular death	246	1.736 (1.275–2.364)	< 0.001			
**Biomarker validation cohorts (plasma)**							
*Beijing validation cohort*							
CECR1 (per 1000 ng/mL decrease)	Cardiovascular death	1,238	3.731 (2.284–6.096)	< 0.001			
CECR1 (per 1000 ng/mL decrease)	Sudden cardiac death	1,238	7.398 (3.319–16.489)	< 0.001	1,002	8.933 (2.906–27.462)	< 0.001
*Xi’an validation cohort*							
CECR1 (per 1000 ng/mL decrease)	Cardiovascular death	402	4.688 (1.818–12.085)	0.001			
CECR1 (per 1000 ng/mL decrease)	Sudden cardiac death	402	4.808 (1.484–15.576)	0.009	258	4.210 (1.313–13.496)	0.016

*CI* confidence interval, *HCM* hypertrophic cardiomyopathy, *HR* hazard ratio

^*^Multivariable models were fitted using a stepwise method with age, sex, symptoms (including chest tightness, chest pain, NYHA class, and syncope), atrial fibrillation, family history, medical history, left atrial diameter, maximal wall thickness, left ventricular (LV) end-diastolic diameter, LV ejection fraction, and LV outflow tract gradient initially included

^†^Sensitivity analysis for the sudden cardiac death endpoint was further performed in the subset of patients with available Holter monitoring data

To explore the molecular links between downregulated *CECR1* expression and increased risk, we next performed Gene Set Enrichment Analysis (GSEA). On the basis of the median stratification, the GSEA results revealed that low *CECR1* expression was associated with significant activation of specific proinflammatory networks. Notably, the IL-17 signalling pathway (normalized enrichment score (NES)=−1.816; adjusted *P* < 0.001; Fig. S3a) and the cytokine‒cytokine receptor interaction pathway (NES =− 1.697; adjusted *P* < 0.001; Fig. S3b) were prominently enriched in patients with lower *CECR1* levels. The negative NES values confirm the inverse relationship between *CECR1* and myocardial inflammatory status.

### Validation of myocardial *CECR1* expression and its correlation with plasma levels

After identifying *CECR1* through transcriptomic screening, we next sought to validate its differential expression in myocardial tissue using experimental approaches and investigated whether myocardial *CECR1* expression changes were reflected in circulating plasma levels.

**Fig. 3:**
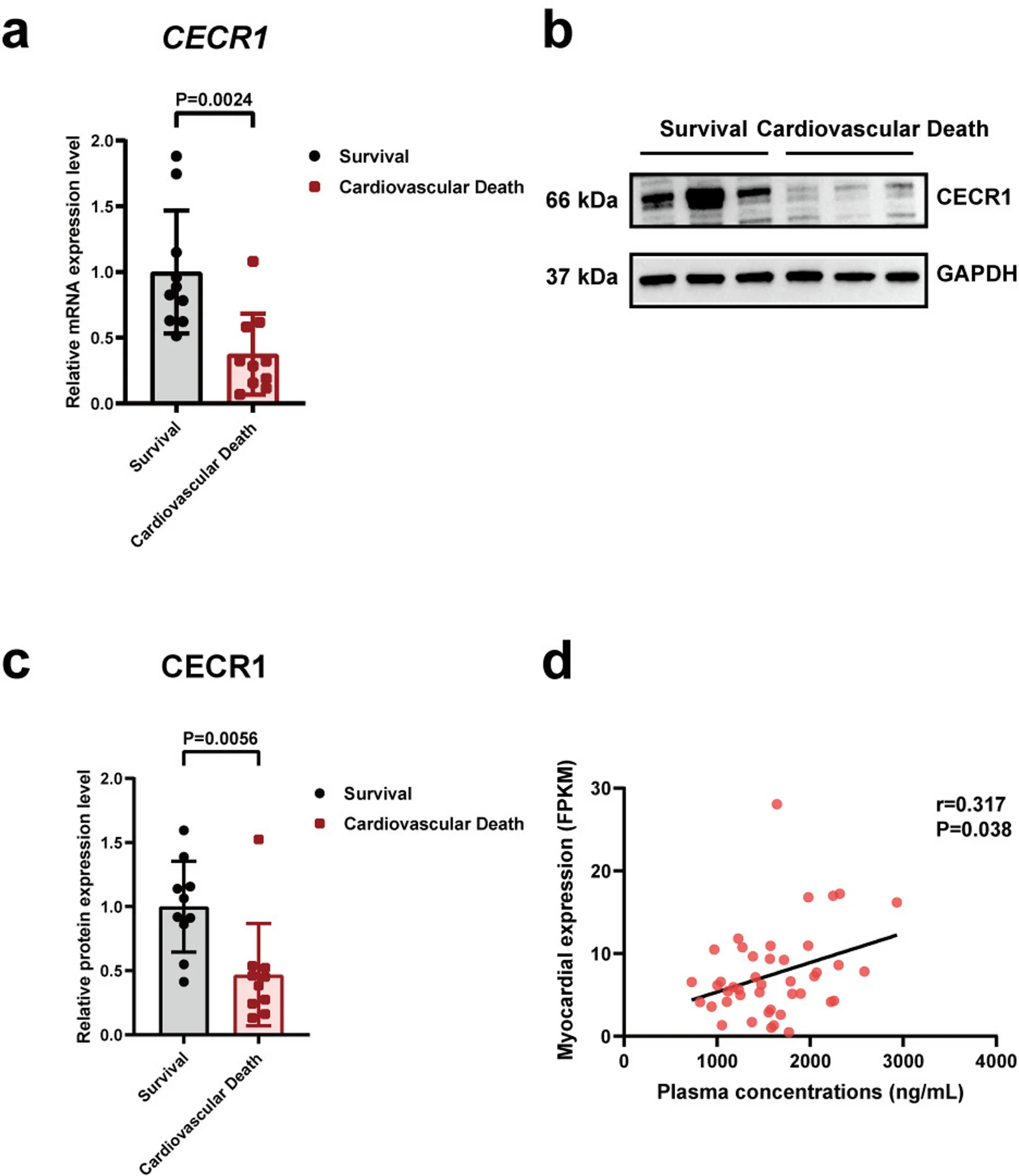
Experimental validation of the correlation between the expression of CECR1 in plasma and its expression in the myocardium. **a** *CECR1* mRNA expression was significantly lower in the cardiovascular death group than in the age- and sex-matched survival group (*n* = 10 per group). **b**, **c** The protein expression of CECR1 was significantly lower in the cardiovascular death group than in the age- and sex-matched survival group (*n* = 10 per group). **d** Correlation between plasma and myocardial CECR1 expression. FPKM, fragments per kilobase of transcript per million mapped reads

To validate the findings from the transcriptomic analysis, we measured CECR1 mRNA and protein expression in myocardial tissues from HCM patients who experienced cardiovascular death and those who survived by quantitative real-time PCR and western blotting, respectively. The results showed that CECR1 mRNA (Fig. 3a) and protein expression (Fig. 3b, c) were significantly lower in the cardiovascular death group than in the age- and sex-matched survival group, further confirming the association between CECR1 expression and prognosis in HCM patients.

To determine whether molecular changes in the diseased heart can be noninvasively detected in the peripheral circulation, we examined the correlation between *CECR1* expression in myocardial tissue and CECR1 protein levels in plasma. For this analysis, paired myocardial and plasma samples were obtained from an overlapping subgroup of 43 patients included in both the discovery cohort and the internal plasma validation cohort (Beijing validation cohort). Age differed slightly between this subgroup of 43 patients and the entire discovery cohort population; however, all the other main clinical characteristics and echocardiographic findings, including sex, symptoms, maximal wall thickness, and LV ejection fraction, were not significantly different (Table S4). We measured plasma CECR1 levels in these patients using an ELISA, and the results revealed a significant, albeit modest, correlation between plasma and myocardial *CECR1* expression (*r* = 0.317; *P* = 0.038; Fig. 3d), suggesting that plasma CECR1 levels, to a certain extent, correspond to those in myocardial tissue.

### Association of plasma CECR1 expression with prognosis in patients with HCM

We next investigated whether circulating CECR1 levels could recapitulate the prognostic signal in the internal plasma validation cohort (Beijing validation cohort). The baseline characteristics of this cohort are summarized in Table S5, and a comparison with the discovery cohort is provided in Table S6. Correlation analyses revealed that plasma CECR1 expression was significantly associated with age, non-sustained ventricular tachycardia (NSVT), maximal wall thickness, the LV outflow tract gradient, and the final conventional SCD risk score (Fig. S4), suggesting its association with the structural and rhythmic risk profile of HCM.

**Fig. 4:**
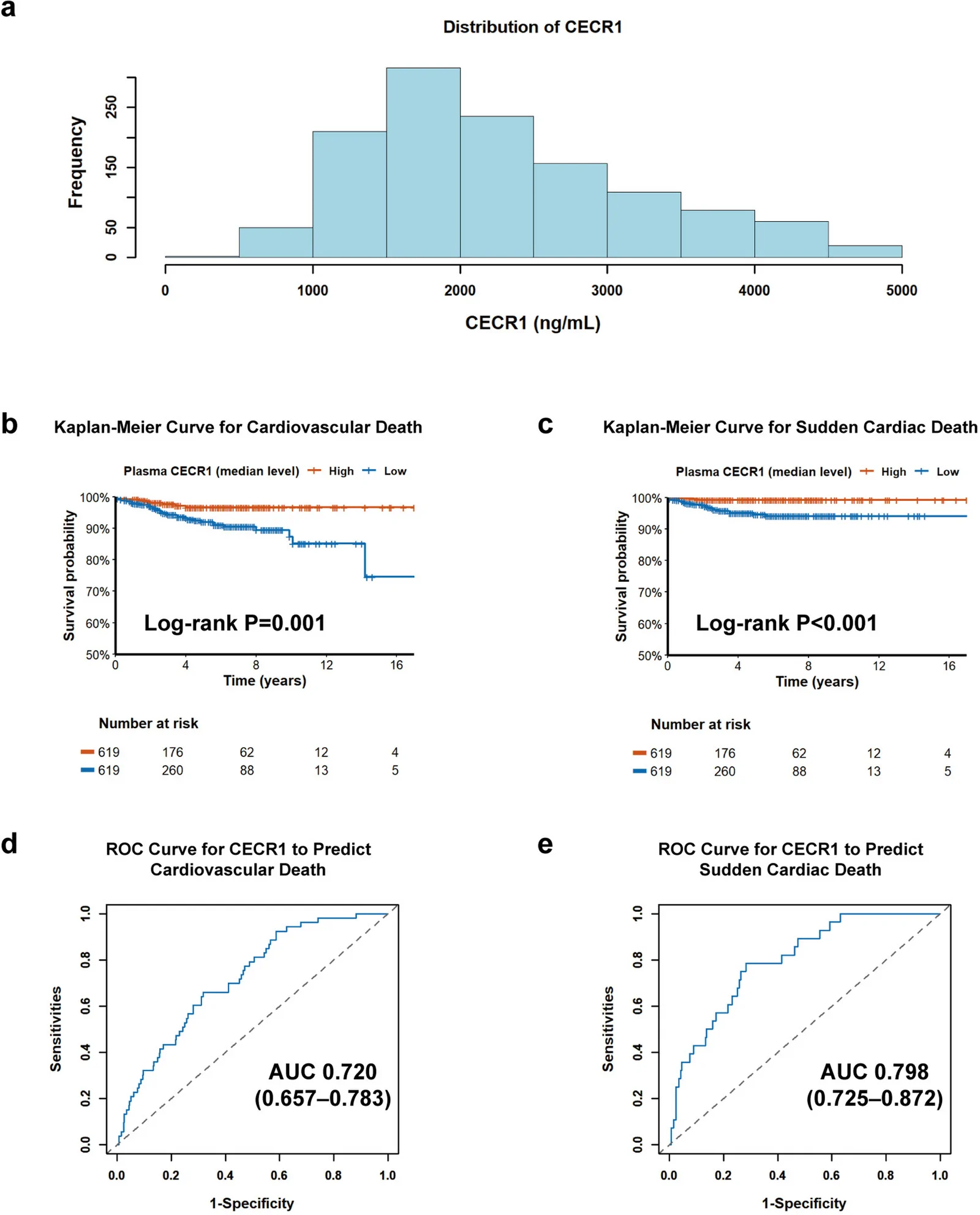
The associations of plasma CECR1 expression with outcomes in the Beijing validation cohort. **a** Distribution of plasma CECR1 levels. **b** Kaplan‒Meier curve for cardiovascular death according to the median level of plasma CECR1 expression. **c** Kaplan‒Meier curve for sudden cardiac death according to the median level of plasma CECR1 expression. **d** ROC curve for the ability of CECR1 to predict cardiovascular death. **e** ROC curve for the ability of CECR1 to predict sudden cardiac death. The analysis of cardiovascular death included 1,238 patients with 53 events, and that of sudden cardiac death included the same cohort with 28 events. AUC, area under the curve; HCM, hypertrophic cardiomyopathy; ROC, receiver operating characteristic

During a median follow-up period of 2.5 (1.5–5.6) years, 53 (4.3%) patients experienced cardiovascular death, and 28 (2.3%) had SCD. The distribution of plasma CECR1 levels in the internal plasma validation cohort is shown in Fig. 4a.

In the entire Beijing validation cohort (*n* = 1,238), multivariable Cox analyses indicated that a lower plasma CECR1 level was independently associated with an increased risk of cardiovascular death (per 1000 ng/mL decrease: adjusted HR 3.731, 95% CI 2.284–6.096, *P* < 0.001) and SCD (per 1000 ng/mL decrease: adjusted HR 7.398, 95% CI 3.319–16.489, *P* < 0.001; Table 1). Kaplan–Meier analyses stratified by the median CECR1 level demonstrated that patients with low CECR1 expression had significantly greater incidences of cardiovascular death (Log-rank *P* = 0.001) and SCD (Log-rank *P* < 0.001; Fig. 4b, c). ROC analysis revealed that plasma CECR1 levels showed good discriminatory performance for cardiovascular death and SCD, with AUCs of 0.720 (95% CI 0.657–0.783) and 0.798 (95% CI 0.725–0.872), respectively (Fig. 4d, e).

Given that NSVT is an established risk marker for SCD, we performed a dedicated sensitivity analysis restricted to patients with complete Holter data (*n* = 1,002) to minimize potential bias due to missing Holter-derived variables in the multivariable Cox analysis of the SCD endpoint. In this complete-case sensitivity analysis, CECR1 remained significantly associated with an increased risk of SCD (per 1000 ng/mL decrease: adjusted HR 8.933, 95% CI 2.906–27.462, *P* < 0.001; Table 1). Together, these findings demonstrated that decreased plasma CECR1 levels identified HCM patients at higher risk of adverse outcomes, supporting its utility as a non-invasive prognostic biomarker.

To further evaluate the robustness of the identified biomarker, we performed stratified Cox regression analyses in obstructive and non-obstructive HCM subgroups. As presented in Table S7, the predictive performance of plasma CECR1 remained stable and significant across both phenotype groups (P for interaction = 0.844 and 0.346 for cardiovascular death and SCD, respectively), suggesting that circulating CECR1 maintains its clinical predictive strength regardless of the patient’s haemodynamic gradient status.

Furthermore, as N-terminal pro-B-type natriuretic peptide (NT-proBNP) and cardiac troponins are known prognostic plasma markers in HCM [9, 10], we evaluated the prognostic performance of CECR1 against these two traditional biomarkers. Our findings indicate that compared with NT-proBNP and high-sensitivity cardiac troponin I, CECR1 has superior predictive capacity (Table S8). Circulating CECR1 levels showed an inverse trend with NT-proBNP and troponin (Fig. S5). In multivariable Cox sensitivity analyses restricted to the 798 patients with both NT-proBNP and troponin measurements available in the Beijing internal validation cohort, CECR1 remained independently associated with both cardiovascular death and sudden cardiac death after further adjustment for these two markers (Table S9), confirming its incremental prognostic value beyond established circulating cardiac markers.

### External validation of the association of plasma CECR1 expression with outcomes

**Fig. 5:**
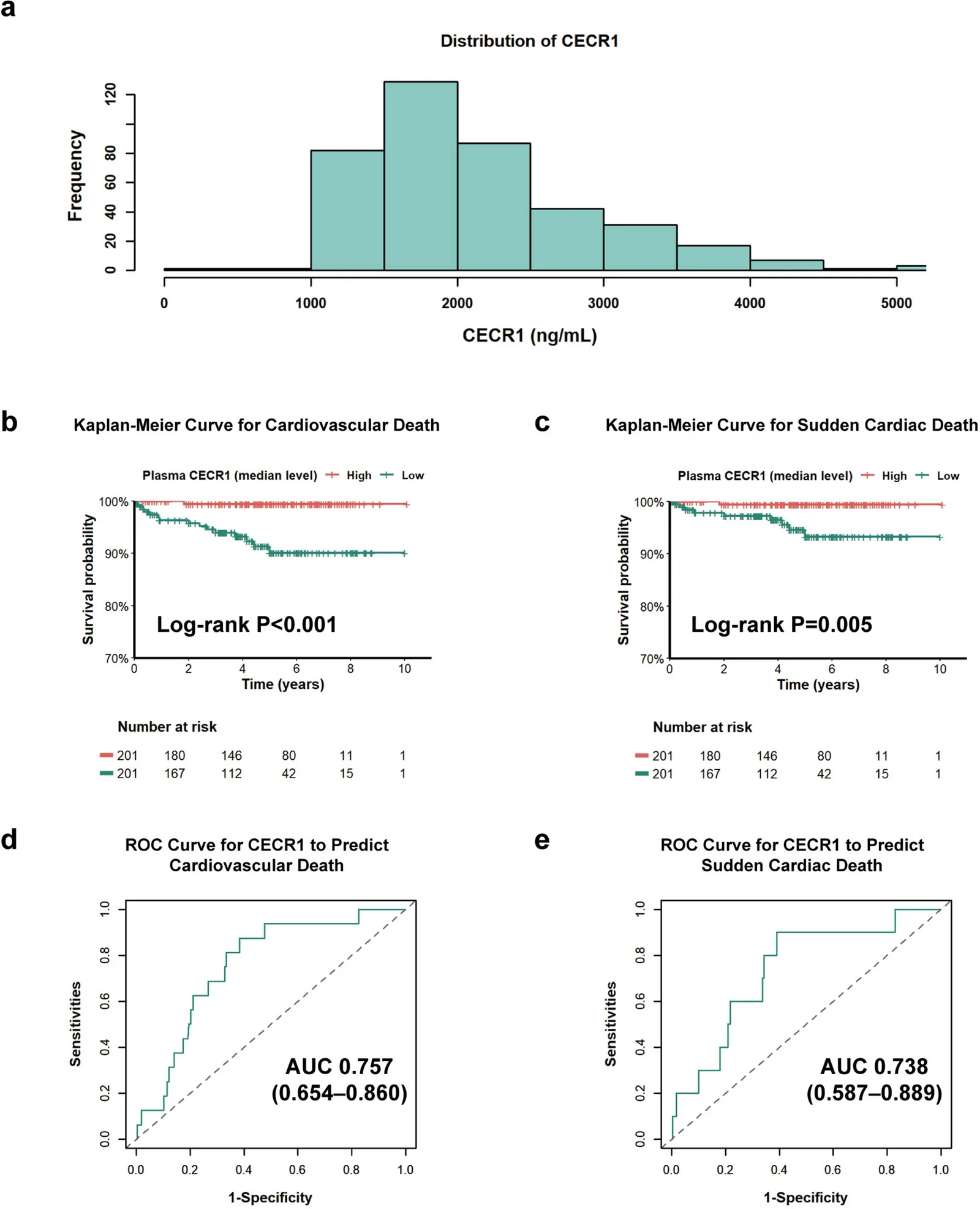
External validation of the associations of plasma CECR1 expression with outcomes. **a** Distribution of plasma CECR1 levels. **b** Kaplan‒Meier curve for cardiovascular death according to the median level of plasma CECR1 expression. **c** Kaplan‒Meier curve for sudden cardiac death according to the median level of plasma CECR1 expression. **d** ROC curve for the ability of CECR1 to predict cardiovascular death. **e** ROC curve for the ability of CECR1 to predict sudden cardiac death. The analysis of cardiovascular death included 402 patients with 16 events, and that of sudden cardiac death included the same cohort with 10 events. AUC, area under the curve; HCM, hypertrophic cardiomyopathy; ROC, receiver operating characteristic

Another independent cohort, the plasma validation cohort from Xijing Hospital, Fourth Military Medical University (Xi’an validation cohort), was used to further validate the prognostic value of plasma CECR1 levels. The baseline characteristics of this cohort are summarized in Table S10. A total of 16 (4.0%) patients experienced cardiovascular death, and 10 (2.5%) had SCD during the median follow-up period of 4.8 (3.3–6.4) years. The distribution of plasma CECR1 levels in the plasma validation cohort was similar to that in the internal plasma validation cohort (Fig. 5a). In the entire Xi’an validation cohort (*n* = 402), multivariable Cox analyses confirmed the associations between the plasma CECR1 level and the risk of cardiovascular death (per 1000 ng/mL decrease: adjusted HR 4.688; 95% CI 1.818–12.085, *P* = 0.001) and SCD (per 1000 ng/mL decrease: adjusted HR 4.808; 95% CI 1.484–15.576, *P* = 0.009; Table 1).

Similarly, lower plasma CECR1 levels were significantly associated with greater incidences of cardiovascular death and SCD. Kaplan‒Meier curves demonstrated clear separation of survival between groups stratified by the median CECR1 level in the plasma validation cohort (Log-rank P for cardiovascular death < 0.001; Log-rank P for SCD = 0.005; Fig. 5b, c). The AUCs of plasma CECR1 for predicting cardiovascular death and SCD were 0.757 (95% CI 0.654–0.860) and 0.738 (95% CI 0.587–0.889), respectively (Fig. 5d, e), indicating reliable discriminatory ability.

In the complete-case sensitivity analysis with complete Holter data (*n* = 258), lower plasma CECR1 levels remained associated with a higher risk of SCD (per 1000 ng/mL decrease: adjusted HR 4.210, 95% CI 1.313–13.496, *P* = 0.016; Table 1). Additionally, the predictive performance of plasma CECR1 remained consistent across both the obstructive and non-obstructive groups (Table S7), which was in line with the findings from the internal plasma cohort. Collectively, the consistent associations observed in this independent cohort support the robustness and generalizability of CECR1 as a prognostic biomarker. Sensitivity analyses excluding procedure-related deaths showed consistent findings. Lower plasma CECR1 levels remained independently associated with cardiovascular death (Table S3), with worse survival (Log-rank *P* < 0.001; Fig. S6a) and an AUC of 0.742 (95% CI 0.624–0.847; Fig. S6b).

### Adding CECR1 improves the SCD prediction model

Plasma CECR1 levels predicted SCD in patients with HCM in the two plasma cohorts; therefore, we next examined whether adding plasma CECR1 expression levels to the HCM Risk-SCD model could enhance its predictive performance.

**Fig. 6:**
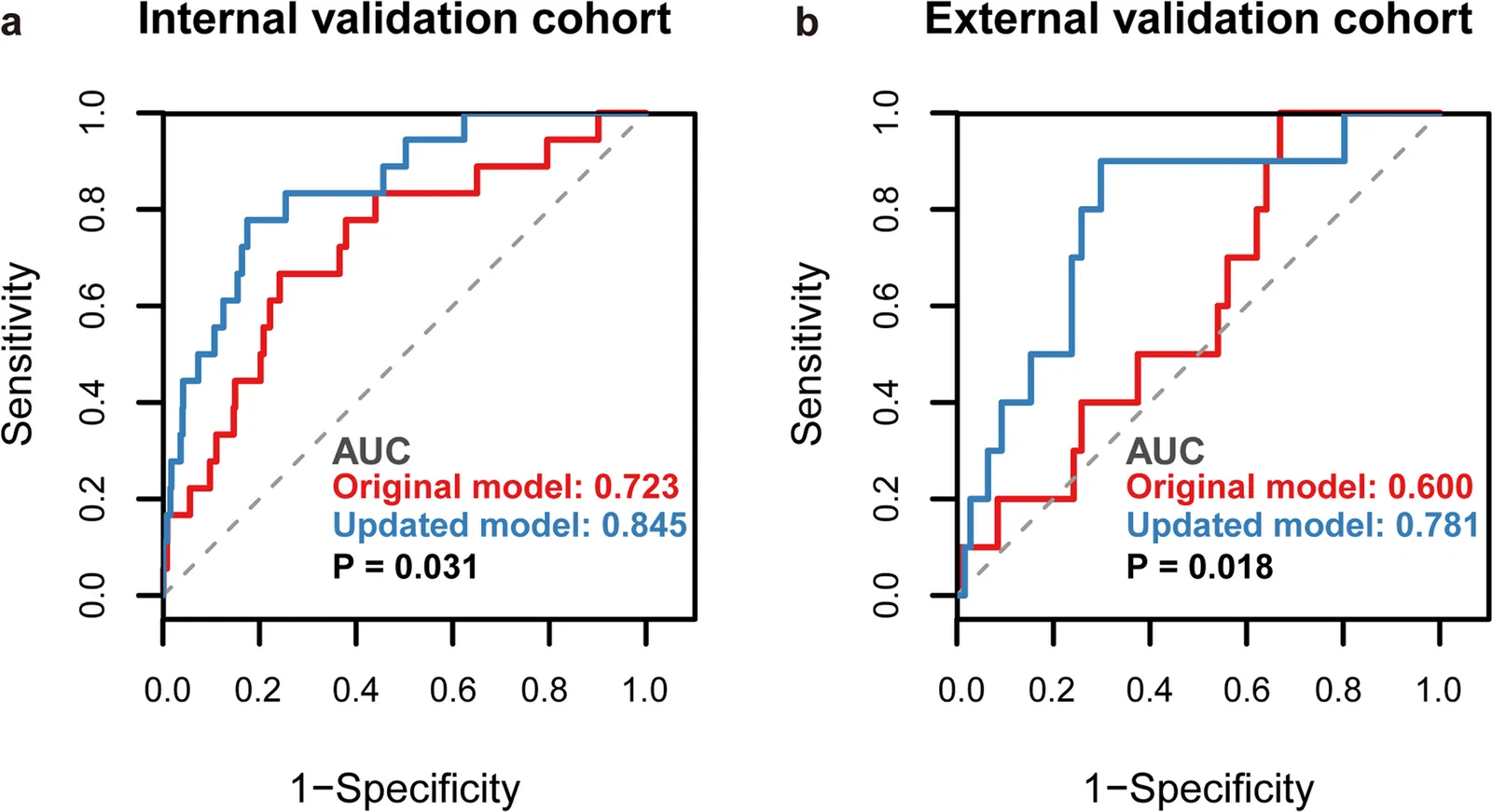
Improvement of the HCM Risk-SCD model. **a** The addition of plasma CECR1 expression to the HCM Risk-SCD model significantly improved the performance of the original model in the Beijing validation cohort (*n* = 1,002 patients with 18 events). **b** Validation of the enhanced model integrating plasma CECR1 expression in the Xi’an validation cohort (*n* = 258 patients with 10 events). AUC, area under the curve; HCM, hypertrophic cardiomyopathy; SCD, sudden cardiac death

We first evaluated this incremental prognostic value within the internal validation cohort. The results revealed that the AUC of the original HCM Risk-SCD model was 0.723 (95% CI 0.598–0.848). Adding plasma CECR1 levels significantly enhanced the predictive performance of the original model, increasing the AUC to 0.845 (*P* = 0.031, Fig. 6a). Furthermore, adding CECR1 yielded a significant continuous net reclassification index (NRI) of 0.994 (95% CI 0.645–1.343, *P* < 0.001) and an integrated discrimination improvement (IDI) of 0.050 (95% CI 0.013–0.087, *P* = 0.008). These values reflect a substantial net improvement in correctly reclassifying patient risk status and enhanced separation of probability distributions between individuals with and without events.

In the external plasma validation cohort, adding CECR1 similarly improved the AUC of the HCM Risk-SCD model from 0.600 to 0.781 (*P* = 0.018) (Fig. 6b), with a continuous NRI of 0.986 (95% CI 0.594–1.377, *P* < 0.001) and an IDI of 0.030 (95% CI 0.007–0.052, *P* = 0.009). Together, these findings suggest that CECR1 may complement the established SCD risk model and improve risk stratification for identifying patients at high risk of SCD.

## Discussion

In this study, we identified CECR1 as a novel prognostic biomarker for HCM by combining myocardial gene expression analysis and circulating protein testing in large HCM cohorts. CECR1 expression was significantly lower in the myocardial tissue of HCM patients who experienced cardiovascular death than in that of patients who survived. Moreover, myocardial and plasma CECR1 levels were correlated, and more importantly, an association between plasma CECR1 levels and prognosis was demonstrated in two independent HCM cohorts, indicating that CECR1 measurement is feasible for the risk stratification of HCM patients.

In myocardial diseases, tissue-level alterations (e.g., myocardial fibrosis and fibrofatty replacement) directly reflect pathological progression and critically determine prognosis [11, 12]. However, compared with conventional analyses of macroscopic and histopathological alterations, transcriptome-derived biomarker profiling offers better resolution for understanding disease mechanisms. Moreover, unlike previous studies that predominantly relied on searching for candidate blood biomarkers using predefined panels (e.g., NT-proBNP and troponins [9, 10]), directly decoding molecular changes in diseased tissues may hold greater potential for biomarker discovery.

Although human myocardial tissue is difficult to access, which limits cardiac biomarker research, HCM presents an opportunity because of its unique septal myectomy procedure, allowing researchers to sequence the resected hypertrophic myocardium and find more specific biomarkers. Nevertheless, myectomy is difficult, posing significant challenges to establishing robust tissue banks with long-term follow-up data. In this study, we included 269 HCM patients who underwent myectomy and myocardial transcriptomic analysis, of whom 246 were successfully followed up for a median duration of 6.8 years. On the basis of these data, we conducted screening for the first time through transcriptome analysis at the myocardial tissue level and revealed that *CECR1* expression was the most powerful prognostic signal in HCM patients.

However, direct detection of gene expression in myocardial tissue is not feasible in clinical practice; therefore, we explored whether plasma CECR1 levels also have prognostic significance. Accordingly, we found a modest but significant correlation between plasma and myocardial *CECR1* levels, suggesting that plasma CECR1 levels, to a certain extent, correspond to those in myocardial tissue. Subsequent investigations in two independent plasma cohorts confirmed an association between plasma CECR1 levels and prognosis in patients with HCM. This concordance between tissue and plasma may be explained by the predominant expression of CECR1 in monocytes and its secretion into the circulation upon monocyte activation [13].

CECR1, also known as ADA2, is a secreted protein that is abundantly expressed in the human heart (data from the Genotype-Tissue Expression Project). In situ hybridization of a human embryo revealed specific CECR1 expression in the outflow tract of the developing heart [14], which aligns with the most commonly affected lesion sites in HCM. Myocardial involvement has been reported in patients with deficiency of CECR1, including dilated cardiomyopathy [15, 16], arrhythmias [16, 17], and myocarditis [17], suggesting the role for CECR1 in myocardial pathophysiology.

The prognostic value of CECR1 may be mediated by multiple mechanisms. HF is often the presenting clinical syndrome in patients with HCM. In the majority of patients, HF in HCM manifests as an HF with preserved ejection fraction (HFpEF) phenotype [18], and HCM patients with HFpEF have been demonstrated to have a worse prognosis [19]. In this study, we observed that patients with low CECR1 levels exhibited more pronounced HF symptoms and markedly elevated NT-proBNP and troponin levels, suggesting that those with low CECR1 expression had a greater HF burden, which may account for the observed poorer prognosis.

Notably, systemic chronic inflammation has been proposed as a central mechanism in the pathophysiology of HFpEF [20]. When the molecular signatures under low-CECR1 conditions were captured, we observed striking activation of downstream proinflammatory cascades, most notably the IL-17 signalling pathway and cytokine–cytokine receptor networks. Emerging evidence has demonstrated that the growth or differentiation factor activity of CECR1 is stronger than the adenosine deamination activity [21, 22]. Specifically, CECR1 regulates macrophage maturation and facilitates the polarization of macrophages towards an anti-inflammatory M2 phenotype [23, 24]. In HCM, chronic myocardial injury accompanied by inflammatory infiltration is a common pathological feature [25, 26]. Low myocardial CECR1 expression thus drives the HCM heart towards a more proinflammatory profile, which can exacerbate myocardial remodelling, impair energy metabolism, and lead to adverse outcomes [27].

Moreover, CECR1 plays a role in maintaining vascular integrity, as its deficiency disrupts the cell junctions of microvascular endothelial cells [28]; such dysregulation is known to contribute to microvascular dysfunction and HFpEF [29]. In addition, a previous study reported the proangiogenic properties of CECR1 [30]. Vasculopathy and attenuated angiogenesis mediated by low myocardial CECR1 expression may cause a decrease in capillary density, disrupting the balance between oxygen supply and demand in the hypertrophic myocardium and resulting in myocardial hypoxia, and microvascular dysfunction potentially contributes to unfavourable outcomes [31, 32].

SCD represents the most catastrophic outcome in patients with HCM [33]. ICD implantation is the only effective approach for preventing SCD; however, a careful, balanced assessment of the potential benefits and risks for each patient is needed. Early and precise identification of patients at high risk for SCD, who may benefit from ICD implantation, is critically important in the management of HCM [34]. The two currently available prediction models have several limitations. The ESC 2014 HCM Risk-SCD model has high specificity but low sensitivity, which may cause the underidentification of high-risk patients. Conversely, the ACC/AHA model has high sensitivity but may result in unnecessary ICD implantation [35]. Furthermore, current prediction models lack the integration of appropriate biomarkers that could enhance risk stratification accuracy. Our screening analysis revealed that myocardial CECR1 is a novel prognostic biomarker in HCM patients, with subsequent validation demonstrating its prognostic utility in plasma. Furthermore, compared with traditional HCM biomarkers, NT-proBNP and troponins, CECR1 exhibited significantly better predictive performance. More importantly, incorporating plasma CECR1 levels into the HCM Risk-SCD model significantly improved its predictive performance. These findings suggest that measuring CECR1 levels could effectively guide the primary prevention of SCD in HCM patients and aid in decision-making regarding ICD implantation.

Several limitations of this study should be acknowledged. First, tissue samples were obtained from a high-volume tertiary surgical center. The distribution of surgical indications at our center may differ from that at other institutions, as surgical management is relatively more proactive in our practice. Therefore, the generalizability and reproducibility of the tissue-based findings should be further evaluated in HCM cohorts from centers with different profiles. Second, owing to factors including medical costs, patient acceptance, and challenges related to long-term device management and post-transplant care, the rates of ICD implantation and heart transplantation remain relatively low in China. Incomplete assessment of SCD risk factors, such as NSVT, may have contributed to the underuse of primary-prevention ICD implantation. Third, the P-value in the screening phase did not reach genome-wide significance, primarily because of the sample size constraint of the discovery cohort and the large number of candidate genes. Although subsequent experiments confirmed the association as a true positive, larger-scale HCM myocardial tissue cohorts are needed to increase the statistical power and more comprehensively define the overall relationship between myocardial gene expression and prognosis in HCM patients. Fourth, not all patients with tissue samples had matching plasma samples. Furthermore, in the correlation analysis between myocardial and plasma CECR1 levels, the correlation, although significant, was modest, suggesting that while plasma CECR1 levels correspond to those in myocardial tissue, they do not fully overlap. The differential information carried by these two sample types requires further mechanistic investigation. Fifth, the prevalence of a positive family history may have been underestimated in our study, as family history was collected on the basis of clinical interviews at admission rather than systematic family screening. Finally, the initial discovery was derived from obstructive HCM patients who underwent surgical myectomy, a patient group that represents a population with more advanced and severe phenotypes, which may introduce potential bias. Future studies should be expanded to include myocardial biopsy cohorts from nonobstructive HCM patients to better reflect the general HCM population.

## Conclusions

This study revealed *CECR1* as a novel prognostic biomarker for HCM through transcriptomic profiling of myocardial tissue. *CECR1* is the first biomarker directly identified from diseased human myocardium in HCM patients. Importantly, plasma CECR1 levels, which correlated significantly with myocardial expression, were strongly associated with both cardiovascular death and SCD. The validation in plasma largely expands the clinical translational potential of this biomarker. Furthermore, the addition of CECR1 to the HCM Risk-SCD model significantly improved model performance, enhancing clinical decision-making for ICD implantation. These findings suggest the potential value of CECR1 assessment for risk stratification and therapeutic decision-making in HCM patients.

## Methods

### Participants and study design

This study included three HCM cohorts. A myocardial discovery cohort was used to identify prognostic biomarkers from myocardial tissues from HCM patients, an internal plasma validation cohort was used to validate the identified biomarkers in HCM plasma, and an external plasma validation cohort was used to further validate the identified biomarkers in an external dataset (Fig. 1). HCM was diagnosed in accordance with the guidelines, defined as otherwise unexplained left ventricular hypertrophy with a maximal ventricular wall thickness ≥ 15 mm, or ≥ 13 mm in individuals with a positive genotype or a confirmed family history of HCM, after excluding loading conditions sufficient to account for the degree of hypertrophy (e.g., uncontrolled hypertension or cardiac valve disease) [5].

#### Myocardial biomarker discovery cohort

The discovery cohort included 269 unrelated patients with HCM who underwent myectomy at Fuwai Hospital, Chinese Academy of Medical Sciences. The inclusion criteria for this cohort were a confirmed diagnosis of HCM and available myocardial tissue collected from septal myectomy. The exclusion criterion was loss to follow-up. Transcriptomic data were obtained from the myocardial tissues of these patients. Patients lost to follow-up (*n* = 23) were excluded; therefore, 246 patients were included in the final discovery analysis. The indications for myectomy in analysed patients with HCM are summarized in Table S11.

#### Beijing internal validation cohort

The Beijing internal validation cohort included 1,339 patients with HCM enrolled from Fuwai Hospital. The inclusion criteria for this cohort were a confirmed diagnosis of HCM and available peripheral plasma samples. The exclusion criterion was loss to follow-up. Plasma samples were collected at enrolment. After patients lost to follow-up were excluded, the final analysis included 1,238 patients.

#### Xi’an external validation cohort

For the Xi’an external validation cohort, 413 patients were enrolled from Xijing Hospital, Fourth Military Medical University. The inclusion criteria for this cohort were a confirmed diagnosis of HCM and available peripheral plasma samples. The exclusion criterion was loss to follow-up. Their plasma samples were collected at enrolment. After the exclusion of patients lost to follow-up, the final analysis included 402 patients.

### Data collection

NSVT data were obtained from 24-h digital Holter monitoring. Because Holter monitoring was not universally performed for all patients, NSVT data are reported only for those who underwent baseline Holter assessment. Similarly, NT-proBNP and troponin levels are reported only for patients with available baseline measurements.

Family history was assessed and recorded in the “Family History” section of the inpatient medical records upon admission to the ward. Specific details were documented, including which family members died of SCD and at what age, as well as which family members received a definite diagnosis of HCM and at what age. Residents were systematically trained to obtain the most accurate family history possible. A positive family history of SCD was defined as a history of SCD at ≤ 50 years of age (definite or suspected to be caused by HCM) in a first-degree or second-degree relative or a history of SCD in multiple third-degree relatives. A positive family history of HCM was defined as the presence of one or more members within three generations of the direct lineage diagnosed with HCM.

### Follow-up and outcomes

The clinical outcomes of HCM patients were obtained through clinic visits or telephone interviews until March 2022 for the myocardial biomarker discovery cohort and the internal plasma validation cohort, and until 2023 for the external plasma validation cohort. If a patient could not be reached directly, we contacted their family members. All the interviews were conducted using predesigned questionnaires to systematically collect outcome information. All reported events underwent independent clinical review and adjudication. Standardized investigator training and blinded questionnaires were implemented to enhance the reliability of outcome ascertainment. The primary endpoint of this study was cardiovascular death, including SCD, HF-related death, stroke-related death, and other cardiovascular deaths. The secondary endpoint was SCD, encompassing unexpected witnessed death occurring within 1 h of symptom onset, regardless of whether ventricular fibrillation was documented, or unexpected nocturnal death without preceding clinical deterioration. Additionally, successful resuscitation following cardiac arrest and appropriate ICD therapy for ventricular tachycardia or ventricular fibrillation were categorized as SCD-equivalent events. The distributions and classifications of these endpoints across the cohorts are provided in Table S12. Detailed information regarding the causes and circumstances of all recorded endpoints is listed in Tables S13–S15.

### mRNA sequencing

Total RNA was isolated from myocardial tissue specimens using an RNeasy Mini kit (Qiagen, Germany). A NanoDrop spectrophotometer was used to evaluate the RNA concentration and purity (Thermo Fisher Scientific, USA), whereas integrity was determined on a Bioanalyzer 2100 system using an RNA Nano 6000 Assay Kit (Agilent Technologies, USA). High-quality samples fulfilling the integrity criterion (RNA integrity number > 7.5) were used for mRNA expression profiling. Sequencing libraries were generated with a KAPA mRNA Hyper Prep Kit (Roche, Switzerland) and subsequently sequenced on a HiSeq X-Ten platform (Illumina, USA) following standard procedures. Raw FASTQ reads were first processed with Trimmomatic version 0.36 to remove adapter sequences. The cleaned reads were then mapped to the hg19 human reference genome using TopHat version 2.1.1. Gene-level read counts were generated with HTSeq version 0.10. Quality control metrics for each sample were evaluated using RSeQC version 2.6.4. The BAM files were analysed with Cufflinks version 2.2.1 to yield fragments per kilobase of transcript per million mapped reads (FPKM) values. The raw FPKM data are included in Table S16.

To identify potential prognostic biomarkers of HCM, multiple Log-rank tests of genes associated with cardiovascular death were performed. Protein-coding genes with an average FPKM > 5 across the cohort were included in the analysis. Prior to analysis, all genes were dichotomized on the basis of their median FPKM values. To thoroughly investigate downstream molecular alterations and functional biological networks, the transcriptomic data were subjected to comprehensive differential expression and functional enrichment analyses. Differential expression analysis was performed using the R package DESeq2 version 1.42.0. GSEA was subsequently performed to identify biological pathways that were systematically disrupted between the designated groups. Statistical significance for GSEA was established using the NES, with an adjusted *P*-value < 0.05 indicating significant enrichment.

### Immunoblot analysis

Tissue samples were retrieved from liquid nitrogen storage and ground under liquid nitrogen. The powdered tissue was lysed in 500 μL of RIPA buffer (Beyotime, China) supplemented with protease and phosphatase inhibitors (Roche, Switzerland) followed by incubation on ice for 60 min. Lysates were centrifuged at 15,000 × g for 30 min at 4 °C, and the resulting supernatants were collected for protein analysis, with a BCA protein assay kit (Thermo Fisher Scientific, USA) used to quantify total protein concentrations. Protein samples were mixed with 5 × SDS‒PAGE loading buffer and denatured for 5 min. Equal amounts of total protein (10 μg per lane) were resolved on 4%–12% polyacrylamide gels (Applygen, China) via electrophoresis and electrotransferred onto methanol-activated polyvinylidene difluoride membranes (Bio-Rad, USA) at 300 mA. After blocking with 5% skim milk for 3 h at room temperature, membranes were incubated overnight at 4 °C with specific primary antibodies (listed in Table S17). Target bands were subsequently detected with corresponding secondary antibodies and visualized using Super-Signal West Femto Maximum Sensitivity Substrate (Thermo Fisher Scientific, USA). Signal intensities of immunoreactive bands were quantified using ImageJ software and normalized to GAPDH as the internal control.

### Real-time quantitative PCR analysis

Total RNA was extracted by adding 1 mL of TRIzol (Ambion, USA) per 50 mg of frozen myocardial tissue and homogenizing it with a homogenizer (MagNALyser, Roche, Switzerland) in specific tubes. Afterwards, 200 μL of trichloromethane was added to the homogenates, which were mixed thoroughly and placed on ice for 30 min. The supernatants were carefully transferred into new tubes after they were centrifuged at 15,000 × g for 30 min at 4 °C, after which 500 μL of isopropanol was added to each tube, and the samples were incubated at −20 °C overnight. RNA was obtained after centrifugation at 15,000 × g for 30 min at 4 °C and washing twice in 75% alcohol. RNA concentration, purity, and integrity were evaluated using the aforementioned methods. Reverse transcription of total RNA was performed using an RT reagent kit (Takara, Japan). Quantitative real-time PCR was carried out with a TB-Green PCR kit (Takara, Japan). The primers used for each gene are listed in Table S18. The relative expression of target genes in various groups was calculated using the 2^−ΔΔCt^ method and normalized to the expression level of *GAPDH*.

### ELISA analysis

The plasma CECR1 level was determined using an enzyme-linked immunosorbent assay (ELISA) kit (NOVUS, USA) following the manufacturer's protocol. Human plasma samples were obtained at enrolment from patients after written consent was obtained, and the samples were stored at −80 °C until use. Prior to the ELISA, the plasma samples were thawed and thoroughly mixed. For CECR1 measurement, plasma samples were diluted 200 × with sample diluent, incubated for 90 min at 37 °C, and then incubated with a biotinylated detection antibody for 60 min and an HRP conjugate for 30 min. Substrate reagent was added sequentially at a constant rate, and after a sufficient reaction time, the stop solution was added in the same manner. The colour intensity was measured using a spectrophotometer at 450 nm (TECAN, Switzerland). The CECR1 level was calculated on the basis of the standard curve and the dilution factor. The reproducibility of the CECR1 ELISA was evaluated. The intra-assay correlation coefficient was 0.955 (95% CI 0.862–0.985, *P* < 0.001). To evaluate inter-operator variation, inter-assay reproducibility was independently tested by two investigators (F.W. and C.L.) at 4-week intervals, yielding a correlation coefficient of 0.844 (95% CI 0.324–0.953, *P* < 0.001).

### Statistical analysis

Continuous measures are reported either as the mean ± standard deviation or as the median with the interquartile range, as appropriate. Categorical measures are presented as numbers with corresponding percentages. Comparisons between groups were performed with the independent-samples Student’s *t* test for normally distributed variables and the Mann–Whitney *U* test otherwise, and the chi-square test for categorical variables. Statistical significance was defined as a two-sided *P*-value < 0.05.

Univariable and multivariable Cox proportional hazards regression models were fitted to examine the association between CECR1 expression and outcomes. Effect estimates are reported as HRs with 95% CIs. The multivariable models were fitted using a stepwise selection approach with age, sex, symptoms (including chest tightness, chest pain, NYHA class, and syncope), atrial fibrillation, family history, medical history, left atrial diameter, maximal wall thickness, LV end-diastolic diameter, LV ejection fraction, and LV outflow tract gradient initially included. Given that NSVT is an established predictor of SCD, a sensitivity analysis was performed among patients with complete Holter monitoring data for the analysis of the SCD endpoint to evaluate the robustness of the association between CECR1 and SCD after accounting for NSVT.

ROC curves were generated to evaluate the predictive performance, and AUCs were calculated. Model improvement analyses were performed exclusively in patients who underwent Holter monitoring for SCD assessment. In the model improvement analysis, the AUCs of the original and updated SCD models, NRI, and IDI were calculated and compared. The original HCM Risk-SCD model was constructed according to guideline recommendations [3].

Statistical analyses and figure generation were conducted in R version 4.3.2 (R Foundation for Statistical Computing, Vienna, Austria).

## Supplementary Information

Supplementary Material 1.

## Data Availability

The datasets generated and/or analysed during the current study are available from the corresponding author on reasonable request after evaluation and agreement by our local ethics committee. Additional data and supplementary files accompanying this manuscript are available as electronic supplementary material. Raw RNA-seq data generated in this study have been deposited in the China National GeneBank Sequence Archive of the China National GeneBank DataBase under accession number CNP0007583.
