# Supplementary material for: Cat eye syndrome chromosome region, candidate 1 is a novel prognostic biomarker in hypertrophic cardiomyopathy
Source: Mol Biomed. 2026 Oct 30;7:234. doi: 10.1186/s43556-026-00648-5 (PMC13722671; doi:10.1186/s43556-026-00648-5)
Supplement: Supplementary file 1 [file 43556_2026_648_MOESM1_ESM.zip › Supplementary Materials.docx]

**Cat Eye Syndrome Chromosome Region, Candidate 1 is a Novel Prognostic Biomarker in Hypertrophic Cardiomyopathy**

Yu Zhang^a,b,†^, Fangfang Wan^b,†^, Chen Li^c^, Bo Wang^d^, Mo Zhang^a^, Minjie Lu^b^, Lianming Kang^a^, Liwen Liu^d,*^, Jizheng Wang^b,*^, Lei Song^a,b,e,*^

^a^Cardiomyopathy Ward, Fuwai Hospital, Chinese Academy of Medical Sciences and Peking Union Medical College, Beijing, China

^b^State Key Laboratory of Cardiovascular Disease, Fuwai Hospital, National Center for Cardiovascular Diseases, Chinese Academy of Medical Sciences and Peking Union Medical College, Beijing, China

^c^Center of Coronary artery diseases, Beijing Anzhen Hospital, Capital Medical University, Beijing, China

^d^Department of Ultrasound, Xijing Hospital, Fourth Military Medical University, Xi'an, Shaanxi, China

^e^National Clinical Research Center of Cardiovascular Diseases, Fuwai Hospital, National Center for Cardiovascular Diseases, Chinese Academy of Medical Sciences and Peking Union Medical College, Beijing, China

^†^These authors contributed equally to the study.

^*^Corresponding authors:

Lei Song, MD, PhD

Email: songlqd@126.com (L.S.)

Jizheng Wang, PhD

Email: [jzwang@hotmail.com](mailto:jzwang@hotmail.com) (J.W.)

Liwen Liu, MD

Email: liuliwen@fmmu.edu.cn (L.L.)

**Supplementary figures**

Figure S1. Flowchart of patient inclusion and exclusion.

Figure S2. Sensitivity analysis of myocardial *CECR1* prognostic value after excluding procedure-related deaths in biomarker discovery cohort.

Figure S3. GSEA of biological pathways associated with myocardial *CECR1* expression.

Figure S4. Correlation analysis between plasma CECR1 expression and clinical parameters of the HCM Risk-SCD model.

Figure S5. Correlation analysis between plasma CECR1 expression and established circulating cardiac biomarkers in Beijing internal validation cohort.

Figure S6. Sensitivity analysis of plasma CECR1 prognostic value after excluding procedure-related deaths in Xi’an external validation cohort.

**Supplementary tables**

Table S1. Baseline characteristics of the discovery cohort.

Table S2. Full list of genes with significant associations with cardiovascular death.

Table S3. Multivariable cox regression analysis of CECR1 and cardiovascular death after excluding perioperative mortality.

Table S4. Comparison of baseline clinical characteristics between the paired sample subgroup and the whole discovery cohort.

Table S5. Baseline characteristics of the Beijing validation cohort stratified by median CECR1 expression.

Table S6. Comparison of baseline clinical characteristics between the discovery cohort and the Beijing validation cohort.

Table S7. Association of CECR1 with endpoints in subgroups of HCM.

Table S8. Predictive efficacy of CECR1 versus established plasma biomarkers.

Table S9. Sensitivity multivariable analysis of plasma CECR1 adjusted for NT-proBNP and troponin in the Beijing internal validation cohort.

Table S10. Baseline characteristics of the Xi'an validation cohort stratified by median CECR1 expression.

Table S11. Indications for surgical myectomy among patients in the discovery cohort.

Table S12. Detailed causes of cardiac death across study cohorts.

Table S13. Detailed causes of cardiovascular death in discovery cohort.

Table S14. Detailed causes of cardiovascular death in the Beijing validation cohort.

Table S15. Detailed causes of cardiovascular death in the Xi'an validation cohort.

Table S16. Raw FPKM data.

Table S17. List of antibodies used in this study.

Table S18. Primer sequences for real-time qPCR assay.

**STROBE Checklist of this study**

**Uncropped Western blot images**

**Supplementary figures**

**Figure S1. Flowchart of patient inclusion and exclusion.**

**Figure S2. Sensitivity analysis of myocardial *CECR1* prognostic value after excluding procedure-related deaths in biomarker discovery cohort.**

(a) Kaplan‒Meier curve for cardiovascular death according to the median level of myocardial *CECR1* expression. (b) ROC curve for the ability of *CECR1* to predict cardiovascular death. The analysis included 246 patients with 12 events.

AUC, area under the curve; ROC, receiver operating characteristic.

**Figure S3. GSEA of biological pathways associated with myocardial *CECR1* expression.**

The GSEA plot demonstrated significant enrichment of the IL-17 signaling pathway (a) and the Cytokine-cytokine receptor interaction pathway (b) in the low myocardial *CECR1* expression group.

GSEA, gene set enrichment analysis; HCM, hypertrophic cardiomyopathy; NES, normalized enrichment score.

**Figure S4. Correlation analysis between plasma CECR1 expression and clinical parameters of the HCM Risk-SCD model.**

The bar chart illustrates the correlation between plasma CECR1 levels and various clinical parameters in patients with HCM. The x-axis represents the -log10(P-value), with the vertical dashed line indicating the threshold for statistical significance (P=0.05).

HCM, hypertrophic cardiomyopathy; LAD, left atrial diameter; LVEDD, left ventricular (LV) end-diastolic diameter; LVEF, LV ejection fraction; LVOT, LV outflow tract; MWT, maximal wall thickness; NSVT, non-sustained ventricular tachycardia; SCD, sudden cardiac death.

**Figure S5. Correlation analysis between plasma CECR1 expression and established circulating cardiac biomarkers in Beijing internal validation cohort.**

(a) Correlation between plasma CECR1 and NT-proBNP, (b) Correlation between plasma CECR1 and hs-cTnI.

hs-cTnI, high-sensitivity cardiac troponin I; NT-proBNP, N-terminal pro-B-type natriuretic peptide.

**Figure S6. Sensitivity analysis of plasma CECR1 prognostic value after excluding procedure-related deaths in Xi’an external validation cohort.**

(a) Kaplan‒Meier curve for cardiovascular death according to the median level of plasma CECR1 expression. (b) ROC curve for the ability of CECR1 to predict cardiovascular death. The analysis included 402 patients with 14 events.

AUC, area under the curve; ROC, receiver operating characteristic.

**Supplementary tables**

| **Table S1. Baseline characteristics of the discovery cohort.** | | |
| --- | --- | --- |
|  | **Available (n)** | **Discovery cohort (n=246)** |
| Age (years) | 246 | 45.7±14.5 |
| Sex (male, n, %) | 246 | 153 (62.2) |
| Chest tightness (n, %) | 246 | 188 (76.4) |
| Chest pain (n, %) | 246 | 118 (48.0) |
| Syncope (n, %) | 246 | 47 (19.1) |
| NYHA class Ⅲ / Ⅳ (n, %) | 246 | 92 (37.4) |
| NT-proBNP (pg/ml) | 196 | 1624.0 (875.9–2823.0) |
| hs-cTnI (ng/ml) | 169 | 0.039 (0.017–0.105) |
| **Arrhythmias** |  |  |
| Atrial fibrillation (n, %) | 246 | 35 (14.2) |
| †NSVT (n, %) | 110 | 14 (12.7) |
| **Family history** |  |  |
| Family history of HCM (n, %) | 246 | 55 (22.4) |
| Family history of SCD (n, %) | 246 | 16 (6.5) |
| **Medical history** |  |  |
| Hypertension (n, %) | 246 | 56 (22.8) |
| Diabetes (n, %) | 246 | 4 (1.6) |
| Coronary artery disease (n, %) | 246 | 34 (13.8) |
| **Imaging parameters** |  |  |
| LAD (mm) | 246 | 44.6±6.9 |
| MWT (mm) | 246 | 24.4±5.1 |
| LVEDD (mm) | 246 | 42.5±5.5 |
| LVEF (%) | 246 | 70.9±6.8 |
| LVOT gradient (mmHg) | 246 | 72 (52–100) |
| †Among the study population, 110 patients underwent baseline 24–hour Holter monitoring. HCM, hypertrophic cardiomyopathy; hs-cTnI, high-sensitivity cardiac troponin I; LAD, left atrial diameter; LVEDD, left ventricular (LV) end-diastolic diameter; LVEF, LV ejection fraction; LVOT, LV outflow tract; MWT, maximal wall thickness; NSVT, non-sustained ventricular tachycardia; NT-proBNP, N-terminal pro-B-type natriuretic peptide; NYHA, New York Heart Association; SCD, sudden cardiac death. | | |

**Table S2. Full list of genes with significant associations with cardiovascular death.**

| **Gene_symbol** | **Log_rank P-value** |
| --- | --- |
| *CECR1* | 9.59272E-05 |
| *EDIL3* | 0.001198586 |
| *ATRX* | 0.003195775 |
| *RBBP6* | 0.003681443 |
| *NEMF* | 0.004006107 |
| *NFIB* | 0.004679056 |
| *NEXN* | 0.004705161 |
| *MPHOSPH8* | 0.004806495 |
| *ADAL* | 0.004809737 |
| *FAM177A1* | 0.005015795 |
| *LPP* | 0.005023359 |
| *CLDN12* | 0.005047244 |
| *PURA* | 0.005075425 |
| *GADD45B* | 0.005137488 |
| *KIF21A* | 0.005356697 |
| *KIDINS220* | 0.005450876 |
| *IDI1* | 0.005602073 |
| *XPR1* | 0.005755247 |
| *HIST1H2BK* | 0.005845841 |
| *ANP32E* | 0.006093919 |
| *RNF103* | 0.006137934 |
| *YAF2* | 0.006244454 |
| *ZADH2* | 0.006297995 |
| *CHKA* | 0.006329839 |
| *IAH1* | 0.006342401 |
| *PCYOX1* | 0.006599287 |
| *BTG2* | 0.00681703 |
| *TMEM59* | 0.006877939 |
| *MPV17L2* | 0.006896035 |
| *CDK11B* | 0.006934081 |
| *NDNL2* | 0.007312041 |
| *AGTRAP* | 0.007331614 |
| *PPP1R15A* | 0.007506206 |
| *GSK3A* | 0.007577362 |
| *TIMM22* | 0.007731334 |
| *PFDN1* | 0.007879414 |
| *RHOQ* | 0.008659164 |
| *PCBD1* | 0.00992739 |
| *HIPK3* | 0.016581669 |
| *NSA2* | 0.01710057 |
| *FAHD1* | 0.018174126 |
| *ARID4B* | 0.018521483 |
| *SREK1* | 0.018649198 |
| *YBX3* | 0.018817795 |
| *EPM2AIP1* | 0.018837986 |
| *PRRC2C* | 0.018950903 |
| *CCDC91* | 0.019031306 |
| *GGNBP2* | 0.01920255 |
| *LARP1B* | 0.01935858 |
| *ABLIM3* | 0.019475277 |
| *ARHGAP5* | 0.019918085 |
| *ESF1* | 0.019968626 |
| *FAM133B* | 0.020594611 |
| *PAM16* | 0.020633562 |
| *USP15* | 0.020925885 |
| *MAGI1* | 0.020935406 |
| *DPY19L2* | 0.021267712 |
| *PAFAH2* | 0.021276991 |
| *ZC3H13* | 0.021382833 |
| *ZNF302* | 0.021445963 |
| *AASS* | 0.021613902 |
| *CFD* | 0.021807946 |
| *MYH11* | 0.021900095 |
| *PNISR* | 0.021945808 |
| *DYNC1LI1* | 0.022104637 |
| *CREBZF* | 0.022112316 |
| *PTMA* | 0.022197532 |
| *CD164* | 0.022236696 |
| *SYF2* | 0.022258004 |
| *GATM* | 0.022480686 |
| *CTNNBIP1* | 0.022564964 |
| *RBM24* | 0.022672011 |
| *CIRH1A* | 0.02284022 |
| *DNAJB5* | 0.022887023 |
| *ARL6IP5* | 0.023045076 |
| *NDUFB11* | 0.023075676 |
| *PRPF4B* | 0.023124967 |
| *BCL7A* | 0.023188523 |
| *IER3IP1* | 0.023197028 |
| *GPX4* | 0.023300975 |
| *AP3S1* | 0.023324019 |
| *IDH1* | 0.023396014 |
| *PHLDB2* | 0.023537465 |
| *PPP1R16A* | 0.023545197 |
| *MIF* | 0.023666826 |
| *UGT2B4* | 0.023669549 |
| *COL21A1* | 0.023693528 |
| *RBM25* | 0.023738095 |
| *EID1* | 0.023878765 |
| *ERGIC2* | 0.023892186 |
| *C5orf24* | 0.023918273 |
| *SKA2* | 0.023962797 |
| *DERA* | 0.024007187 |
| *TMEM56* | 0.024195886 |
| *ECD* | 0.024278807 |
| *HAUS4* | 0.024456387 |
| *CERS2* | 0.024608281 |
| *ANKRD23* | 0.024617121 |
| *RP9* | 0.024841389 |
| *SSFA2* | 0.024907166 |
| *FNDC3B* | 0.02490799 |
| *MRPL32* | 0.024931372 |
| *ZYX* | 0.024994418 |
| *MAP4K3* | 0.02502882 |
| *TAF1D* | 0.025034177 |
| *SUB1* | 0.025102001 |
| *SAT1* | 0.025140548 |
| *NES* | 0.025285924 |
| *XIRP1* | 0.02536386 |
| *MAP2K3* | 0.025376175 |
| *EXOSC1* | 0.025388494 |
| *TMEM55A* | 0.02553346 |
| *CWC27* | 0.02555728 |
| *MYH9* | 0.025645941 |
| *APEX1* | 0.025675312 |
| *ZCRB1* | 0.025729318 |
| *RPS15* | 0.025810621 |
| *TRIB1* | 0.0258887 |
| *NSRP1* | 0.025937414 |
| *PCNP* | 0.026017365 |
| *BAG3* | 0.026038425 |
| *CMBL* | 0.026085855 |
| *CDK2AP1* | 0.02617517 |
| *MYL9* | 0.02621057 |
| *C17orf49* | 0.026281758 |
| *DNAJB9* | 0.026317802 |
| *DDX21* | 0.026480038 |
| *VEGFA* | 0.02648772 |
| *CCNK* | 0.026596748 |
| *TIAF1* | 0.026676451 |
| *HSBP1* | 0.026676686 |
| *TMEM71* | 0.026763153 |
| *PPIG* | 0.026835919 |
| *CDK2AP2* | 0.026903802 |
| *APOL3* | 0.026930616 |
| *HSP90AA1* | 0.026987304 |
| *MEF2BNB* | 0.027095875 |
| *ADIPOR1* | 0.027257497 |
| *MLPH* | 0.027314818 |
| *DLGAP4* | 0.027343117 |
| *ZC3H15* | 0.027432054 |
| *CDC37L1* | 0.027640168 |
| *ZNF688* | 0.027643963 |
| *PPM1B* | 0.027700573 |
| *MYOZ2* | 0.027719126 |
| *EIF2S2* | 0.027749423 |
| *AGTPBP1* | 0.027817822 |
| *AMOTL1* | 0.02783276 |
| *CDC42EP3* | 0.027837929 |
| *CALD1* | 0.02784229 |
| *FGFR1OP2* | 0.027845149 |
| *SLC9A1* | 0.027892072 |
| *HNRNPH2* | 0.027991023 |
| *ARL5A* | 0.028095993 |
| *MKRN2* | 0.028102982 |
| *SET* | 0.028152721 |
| *GGT7* | 0.02816044 |
| *DMPK* | 0.028205948 |
| *SNN* | 0.028254088 |
| *SLC38A2* | 0.028270879 |
| *PLA2G5* | 0.028279805 |
| *RGS3* | 0.028382695 |
| *RPL21* | 0.028442161 |
| *COMT* | 0.02844704 |
| *PELP1* | 0.028571781 |
| *CAP2* | 0.028641285 |
| *TTC38* | 0.0286823 |
| *RUSC2* | 0.028780074 |
| *PKIG* | 0.028899172 |
| *RAB11FIP5* | 0.028907466 |
| *SPPL2A* | 0.029018613 |
| *RNF44* | 0.029066147 |
| *INPP5K* | 0.029080686 |
| *DNASE1L1* | 0.029102632 |
| *POPDC3* | 0.029112597 |
| *RAI2* | 0.029365762 |
| *SRA1* | 0.029434523 |
| *CCND2* | 0.029558547 |
| *DTNA* | 0.029705048 |
| *PXN* | 0.029784177 |
| *MRPS16* | 0.029821959 |
| *FKBP4* | 0.030015805 |
| *RNF146* | 0.030030533 |
| *TRIM55* | 0.030137921 |
| *CISD1* | 0.03018321 |
| *PDCD10* | 0.030187323 |
| *FABP4* | 0.030216555 |
| *COMMD8* | 0.030220956 |
| *AGPAT1* | 0.03023382 |
| *SATB1* | 0.030256961 |
| *RPS29* | 0.03051608 |
| *WBSCR22* | 0.030543218 |
| *ARL2* | 0.030579958 |
| *UFSP2* | 0.030593 |
| *POLR2J* | 0.03060416 |
| *P4HTM* | 0.030640943 |
| *PTP4A1* | 0.030651887 |
| *MED21* | 0.030673307 |
| *PLEKHA7* | 0.030737316 |
| *PTGR2* | 0.030778862 |
| *PFN2* | 0.030910332 |
| *SAP18* | 0.031374826 |
| *PRKACA* | 0.031441643 |
| *ANKRD1* | 0.031590567 |
| *C9orf78* | 0.031611635 |
| *ARHGEF10L* | 0.031617664 |
| *AKT1* | 0.031666708 |
| *HARS* | 0.031688687 |
| *GSPT1* | 0.031708929 |
| *BRD4* | 0.031784368 |
| *BPGM* | 0.031844816 |
| *PUM1* | 0.03186558 |
| *TMEM251* | 0.031989373 |
| *FAM220A* | 0.03205922 |
| *DNAJB11* | 0.032111086 |
| *PSMD13* | 0.032187709 |
| *DNAJB1* | 0.032318307 |
| *PRMT1* | 0.032372653 |
| *TNFAIP1* | 0.032440997 |
| *ST6GALNAC4* | 0.03244154 |
| *C1orf198* | 0.03254967 |
| *PLCXD3* | 0.032550893 |
| *CDC26* | 0.032588421 |
| *QDPR* | 0.032638722 |
| *COPS4* | 0.032862278 |
| *C11orf68* | 0.032871782 |
| *H2AFY* | 0.03301237 |
| *CTSZ* | 0.03301457 |
| *TMEM256* | 0.033312795 |
| *SPATS2L* | 0.033486894 |
| *RRAS* | 0.033554222 |
| *CLIP4* | 0.033582498 |
| *CETN2* | 0.033612006 |
| *NFYB* | 0.033658647 |
| *S100A13* | 0.03380446 |
| *GTPBP8* | 0.034209906 |
| *PIP5K1C* | 0.034547495 |
| *RBPMS2* | 0.034682355 |
| *TMA7* | 0.035616509 |
| *FN3K* | 0.0356192 |
| *BCL2L1* | 0.0357606 |
| *MAD2L2* | 0.035766391 |
| *HNRNPH3* | 0.036301641 |
| *SF1* | 0.036578316 |
| *CUEDC2* | 0.036651038 |
| *PRDX2* | 0.037333291 |
| *CASZ1* | 0.0376714 |
| *PLOD3* | 0.038182326 |
| *PSMC4* | 0.038515246 |
| *MRPL18* | 0.038570798 |
| *CDKN1A* | 0.03935543 |
| *ANXA11* | 0.040900115 |
| *SLC6A8* | 0.041222859 |
| *PLA2G16* | 0.042180933 |
| *POR* | 0.042207065 |
| *C16orf72* | 0.042915185 |
| *VTN* | 0.043568712 |
| *DYNLRB1* | 0.044742232 |
| *LPIN3* | 0.047079108 |
| *HIBCH* | 0.047899036 |

| **Table S3. Multivariable cox regression analysis of CECR1 and cardiovascular death after excluding perioperative mortality.** | | | | | |
| --- | --- | --- | --- | --- | --- |
| **Cohort** | | **Number of patients** | **Number  of events** | *****Adjusted HR (95% CI) | *****Adjusted P–value |
| Biomarker discovery cohort | *CECR1* (per 1 FPKM decrease) | 246 | 12 | 1.554 (1.209–1.997) | <0.001 |
| Xi'an validation cohort | CECR1 (per 1000 ng/mL decrease) | 402 | 14 | 6.429 (2.331–17.735) | <0.001 |
| ^*^Multivariable models were fitted using a stepwise method with age, sex, symptoms (including chest tightness, chest pain, NYHA class, and syncope), atrial fibrillation, family history, medical history, left atrial diameter, maximal wall thickness, left ventricular (LV) end-diastolic diameter, LV ejection fraction, and LV outflow tract gradient initially included. | | | | | |

| **Table S4. Comparison of baseline clinical characteristics between the paired sample subgroup and the whole discovery cohort.** | | | | |
| --- | --- | --- | --- | --- |
|  | **Available (n)** | **Subgroup with paired samples  (n=43)** | **Whole discovery cohort (n=246)** | **P-value** |
| Age (years) | 43/246 | 50.3±14.2 | 45.7±14.5 | 0.042 |
| Sex (male, n, %) | 43/246 | 27 (62.8) | 153 (62.2) | 0.978 |
| Syncope (n, %) | 43/246 | 9 (20.9) | 47 (19.1) | 0.780 |
| Chest tightness (n, %) | 43/246 | 28 (65.1) | 188 (76.4) | 0.129 |
| Chest pain (n, %) | 43/246 | 16 (37.2) | 118 (48.0) | 0.246 |
| NYHA class Ⅲ / Ⅳ (n, %) | 43/246 | 12 (27.9) | 92 (37.4) | 0.232 |
| NT-proBNP (pg/ml) | 23/196 | 2425.0 (1056.6–4018.7) | 1624.0 (875.9–2823.0) | 0.062 |
| hs-cTnI (ng/ml) | 21/169 | 0.053 (0.016–0.154) | 0.039 (0.017–0.105) | 0.548 |
| **Arrhythmias** |  |  |  |  |
| Atrial fibrillation (n, %) | 43/246 | 6 (14.0) | 35 (14.2) | 1.000 |
| NSVT (n, %) | 10/110 | 3 (30.0) | 14 (12.7) | 0.151 |
| **Family history** | 43/246 |  |  |  |
| Family history of HCM (n, %) | 43/246 | 12 (27.9) | 55 (22.4) | 0.561 |
| Family history of SCD (n, %) | 43/246 | 2 (4.7) | 16 (6.5) | 1.000 |
| **Medical history** |  |  |  |  |
| Hypertension (n, %) | 43/246 | 14 (32.6) | 56 (22.8) | 0.114 |
| Diabetes (n, %) | 43/246 | 0 (0.0) | 4 (1.6) | 1.000 |
| Coronary artery disease (n, %) | 43/246 | 5 (11.6) | 34 (13.8) | 0.813 |
| **Imaging parameters** |  |  |  |  |
| LAD (mm) | 43/246 | 44.6±6.8 | 44.6±6.9 | 0.906 |
| MWT (mm) | 43/246 | 24.2±5.6 | 24.4±5.1 | 0.783 |
| LVEDD (mm) | 43/246 | 43.0±5.0 | 42.5±5.5 | 0.507 |
| LVEF (%) | 43/246 | 70.0±5.4 | 70.9±6.8 | 0.462 |
| LVOT gradient (mmHg) | 43/246 | 84 (55–110) | 72 (52–100) | 0.157 |
| HCM, hypertrophic cardiomyopathy; hs-cTnI, high-sensitivity cardiac troponin I; LAD, left atrial diameter; LVEDD, left ventricular (LV) end-diastolic diameter; LVEF, LV ejection fraction; LVOT, LV outflow tract; MWT, maximal wall thickness; NSVT, non-sustained ventricular tachycardia; NT-proBNP, N-terminal pro-B-type natriuretic peptide; NYHA, New York Heart Association; SCD, sudden cardiac death. | | | | |

| **Table S5. Baseline characteristics of the Beijing validation cohort stratified by median CECR1 expression.** | | | | | | | | |
| --- | --- | --- | --- | --- | --- | --- | --- | --- |
|  | **Available (n)** | | **Low plasma CECR1 expression  (n=619)** | | **High plasma CECR1 expression  (n=619)** | | **P-value** | |
| Age (years) | 1,238 | | 45.8±14.0 | | 50.2±14.5 | | 0.767 | |
| Sex (male, n, %) | 1,238 | | 396 (64.0) | | 402 (64.9) | | <0.001 | |
| Syncope (n, %) | 1,238 | | 75 (12.1) | | 81 (13.1) | | 0.669 | |
| Chest tightness (n, %) | 1,238 | | 374 (60.4) | | 377 (60.9) | | 0.907 | |
| Chest pain (n, %) | 1,238 | | 184 (29.7) | | 169 (27.3) | | 0.378 | |
| NYHA class Ⅲ / Ⅳ (n, %) | 1,238 | | 157 (25.4) | | 118 (19.1) | | 0.008 | |
| NT–proBNP (pg/ml) | 1,031 | | 835.2 (423.0–1833.0) | | 702.0 (303.9–1612.0) | | 0.005 | |
| hs–cTnI (ng/ml) | 955 | | 0.026 (0.010–0.712) | | 0.020 (0.008–0.056) | | 0.039 | |
| **Therapy** | 1,238 | |  | |  | |  | |
| Septal reduction therapy (n, %) | 1,238 | | 99 (16.0) | | 58 (9.4) | | <0.001 | |
| ICD implantation (n, %) | 1,238 | | 14 (2.3) | | 13 (2.1) | | 0.846 | |
| **Arrhythmias** |  | |  | |  | |  | |
| Atrial fibrillation (n, %) | 1,238 | | 99 (16.0) | | 120 (19.4) | | 0.136 | |
| †NSVT (n, %) | 1,002 | | 44 (9.1) | | 82 (15.7) | | 0.002 | |
| **Family history** |  | |  | |  | |  | |
| Family history of HCM (n, %) | 1,238 | | 117 (18.9) | | 104 (16.8) | | 0.373 | |
| Family history of SCD (n, %) | 1,238 | | 80 (12.9) | | 52 (8.4) | | 0.013 | |
| **Medical history** |  | |  | |  | |  | |
| Hypertension (n, %) | 1,238 | | 180 (29.1) | | 245 (39.6) | | <0.001 | |
| Diabetes (n, %) | 1,238 | | 37 (6.0) | | 48 (7.8) | | 0.261 | |
| Coronary artery disease (n, %) | 1,238 | | 46 (7.4) | | 81 (13.1) | | 0.001 | |
| **Imaging parameters** |  | |  | |  | |  | |
| LAD (mm) | 1,238 | | 40.9±6.2 | | 41.3±6.5 | | 0.285 | |
| MWT (mm) | 1,238 | | 23.1±5.9 | | 22.2±6.3 | | 0.010 | |
| LVEDD (mm) | 1,238 | | 44.1±6.1 | | 45.1±6.4 | | 0.003 | |
| LVEF (%) | 1,238 | | 66.9±7.6 | | 65.7±8.9 | | 0.010 | |
| LVOT gradient (mmHg) | 1,238 | | 16 (7–64) | | 12 (7–49) | | 0.006 | |
| †Among the study population, 1,002 patients underwent baseline 24–hour Holter monitoring. HCM, hypertrophic cardiomyopathy; hs-cTnI, high-sensitivity cardiac troponin I; ICD, implantable cardioverter defibrillator; LAD, left atrial diameter; LVEDD, left ventricular (LV) end-diastolic diameter; LVEF, LV ejection fraction; LVOT, LV outflow tract; MWT, maximal wall thickness; NSVT, non-sustained ventricular tachycardia; NT-proBNP, N-terminal pro-B-type natriuretic peptide; NYHA, New York Heart Association; SCD, sudden cardiac death. | | | | | | | | |
| **Table S6. Comparison of baseline clinical characteristics between the discovery cohort and the Beijing validation cohort.** | | | | | | | | |
|  | | **Available (n)** | | **Discovery cohort (n=246)** | | **Beijing validation cohort (n=1238)** | | **P-value** |
| Age (years) | | 246/1,238 | | 45.7±14.5 | | 48.0±14.4 | | 0.010 |
| Sex (male, n, %) | | 246/1,238 | | 153 (62.2) | | 798 (64.5) | | 0.663 |
| Syncope (n, %) | | 246/1,238 | | 47 (19.1) | | 156 (12.6) | | 0.008 |
| Chest tightness (n, %) | | 246/1,238 | | 188 (76.4) | | 751 (60.7) | | <0.001 |
| Chest pain (n, %) | | 246/1,238 | | 118 (48.0) | | 353 (28.5) | | <0.001 |
| NYHA class Ⅲ / Ⅳ (n, %) | | 246/1,238 | | 92 (37.4) | | 275 (22.2) | | <0.001 |
| NT–proBNP (pg/ml) | | 196/1,031 | | 1624.0 (875.9–2823.0) | | 788.9 (357.0–1722.0) | | <0.001 |
| hs-cTnI (ng/ml) | | 169/955 | | 0.039 (0.017–0.105) | | 0.023 (0.009–0.063) | | <0.001 |
| **Arrhythmias** | |  | |  | |  | |  |
| Atrial fibrillation (n, %) | | 246/1,238 | | 35 (14.2) | | 219 (17.7) | | 0.191 |
| †NSVT (n, %) | | 110/1,002 | | 14 (12.7) | | 126 (12.6) | | 1.000 |
| **Family history** | |  | |  | |  | |  |
| Family history of HCM (n, %) | | 246/1,238 | | 55 (22.4) | | 221 (17.9) | | 0.060 |
| Family history of SCD (n, %) | | 246/1,238 | | 16 (6.5) | | 132 (10.7) | | 0.048 |
| **Medical history** | |  | |  | |  | |  |
| Hypertension (n, %) | | 246/1,238 | | 56 (22.8) | | 425 (34.3) | | <0.001 |
| Diabetes (n, %) | | 246/1,238 | | 4 (1.6) | | 85 (6.9) | | <0.001 |
| Coronary artery disease (n, %) | | 246/1,238 | | 34 (13.8) | | 127 (10.3) | | 0.115 |
| **Imaging parameters** | |  | |  | |  | |  |
| LAD (mm) | | 246/1,238 | | 44.6±6.9 | | 41.1±6.4 | | <0.001 |
| MWT (mm) | | 246/1,238 | | 24.4±5.1 | | 22.6±6.1 | | <0.001 |
| LVEDD (mm) | | 246/1,238 | | 42.5±5.5 | | 44.6±6.3 | | <0.001 |
| LVEF (%) | | 246/1,238 | | 70.9±6.8 | | 66.3±8.3 | | <0.001 |
| LVOT gradient (mmHg) | | 246/1,238 | | 72 (52–100) | | 13 (7–54) | | <0.001 |
| HCM, hypertrophic cardiomyopathy; hs-cTnI, high-sensitivity cardiac troponin I; LAD, left atrial diameter; LVEDD, left ventricular (LV) end-diastolic diameter; LVEF, LV ejection fraction; LVOT, LV outflow tract; MWT, maximal wall thickness; NSVT, non-sustained ventricular tachycardia; NT-proBNP, N-terminal pro-B-type natriuretic peptide; NYHA, New York Heart Association; SCD, sudden cardiac death. | | | | | | | | |

| **Table S7. Association of CECR1 with endpoints in subgroups of HCM.** | | | | | | |
| --- | --- | --- | --- | --- | --- | --- |
|  | **Primary endpoint** | | | **Secondary endpoint** | | |
|  | **Cardiovascular death** | | | **SCD** | | |
|  | ***Adjusted HR (95% CI)** | ***Adjusted P–value** | **P for interaction** | ***Adjusted HR**  **(95% CI)** | ***Adjusted P–value** | **P for interaction** |
| **Beijing validation cohort** | |  |  |  |  |  |
| Non–obstructive HCM (n=784)  CECR1 (per 1000 ng/mL decrease) | 4.837 (2.466–9.488) | <0.001 | 0.844 | 5.188 (1.898–14.179) | 0.001 | 0.346 |
| Obstructive HCM (n=454)  CECR1 (per 1000 ng/mL decrease) | 3.460 (1.452-8.243) | 0.005 |  | 10.111 (2.364–43.249) | 0.002 |  |
| **Xi’an validation cohort** | |  |  |  |  |  |
| Non–obstructive HCM (n=210)  CECR1 (per 1000 ng/mL decrease) | 24.816 (4.477–137.554) | <0.001 | 0.287 | 90.398 (3.613–2261.932) | 0.006 | 0.104 |
| Obstructive HCM (n=192)  CECR1 (per 1000 ng/mL decrease) | 35.988 (2.884–449.014) | 0.005 |  | 67.202 (1.583–2852.771) | 0.028 |  |
| *Multivariable models were fitted using a stepwise method with age, sex, symptoms, atrial fibrillation, family history, medical history, left atrial diameter, maximal wall thickness, left ventricular (LV) end-diastolic diameter, LV ejection fraction, and LV outflow tract gradient initially included.  CI, confidence interval; HCM, hypertrophic cardiomyopathy; HR, hazard ratio; SCD, sudden cardiac death. | | | | | | |

| **Table S8. Predictive efficacy of CECR1 versus established plasma biomarkers.** | | | | |
| --- | --- | --- | --- | --- |
|  | **Primary endpoint** | | **Secondary endpoint** | |
|  | **Cardiovascular death** | | **SCD** | |
|  | **AUC (95% CI)** | ***P-value** | **AUC (95% CI)** | ***P-value** |
| **CECR1 vs. NT-proBNP** (n=1,033) |  |  |  |  |
| CECR1 | 0.798 (0.742–0.853) | 0.514 | 0.864 (0.813–0.914) | 0.037 |
| NT-proBNP | 0.772 (0.706–0.837) |  | 0.764 (0.672–0.855) |  |
| **CECR1 vs. hs-cTnI** (n=814) |  |  |  |  |
| CECR1 | 0.819 (0.760–0.879) | 0.008 | 0.862 (0.800–0.923) | 0.014 |
| hs-cTnI | 0.716 (0.648–0.784) |  | 0.747 (0.670–0.823) |  |
| *AUC comparison was performed using the DeLong test. AUC, area under the curve; CI, confidence interval; hs-cTnI, high-sensitivity cardiac troponin I; NT-proBNP, N-terminal pro-B-type natriuretic peptide; SCD, sudden cardiac death. | | | | |

| **Table S9. Sensitivity multivariable analysis of plasma CECR1 adjusted for NT-proBNP and troponin in the Beijing internal validation cohort.** | | | | | |
| --- | --- | --- | --- | --- | --- |
|  | **Endpoint** | **^#^Number of patients** | **Number of events** | ***Adjusted HR**  **(95% CI)** | ***Adjusted P-value** |
| CECR1 (per 1000 ng/mL decrease) | Cardiovascular death | 798 | 36 | 3.103 (1.774–5.427) | <0.001 |
| CECR1 (per 1000 ng/mL decrease) | Sudden cardiac death | 798 | 21 | 5.636 (2.047–15.517) | <0.001 |
| ^*^Multivariable models were fitted using a stepwise method with NT-proBNP, hs-cTnI, age, sex, symptoms (including chest tightness, chest pain, NYHA class, and syncope), atrial fibrillation, family history, medical history, left atrial diameter, maximal wall thickness, left ventricular (LV) end-diastolic diameter, LV ejection fraction, and LV outflow tract gradient initially included. ^#^ The analysis was restricted to 798 patients with available data for bothNT-proBNP and troponin in the Beijing internal validation cohort. | | | | | |

| **Table S10. Baseline characteristics of the Xi'an validation cohort stratified by median CECR1 expression.** | | | | |
| --- | --- | --- | --- | --- |
|  | **Available (n)** | **Low plasma CECR1 expression  (n=201)** | **High plasma CECR1 expression  (n=201)** | **P-value** |
| Age (years) | 402 | 45.4±14.9 | 45.8±14.7 | 0.780 |
| Sex (male, n, %) | 402 | 119 (59.2) | 142 (70.6) | 0.021 |
| Chest tightness (n, %) | 402 | 141 (70.1) | 124 (61.7) | 0.092 |
| Chest pain (n, %) | 402 | 113 (56.2) | 88 (43.8) | 0.017 |
| Syncope (n, %) | 402 | 46 (22.9) | 43 (21.4) | 0.810 |
| NYHA class Ⅲ / Ⅳ (n, %) | 402 | 41 (20.4) | 38 (19.5) | 0.881 |
| **Therapy** |  |  |  |  |
| Septal reduction therapy (n, %) | 402 | 2 (1.0) | 4 (2.0) | 0.411 |
| ICD implantation (n, %) | 402 | 6 (3.0) | 5 (2.5) | 0.760 |
| **Arrhythmias** |  |  |  |  |
| Atrial fibrillation (n, %) | 402 | 9 (4.5) | 12 (6.0) | 0.654 |
| †NSVT (n, %) | 258 | 19 (14.4) | 22 (17.5) | 0.501 |
| **Family history** |  |  |  |  |
| Family history of HCM (n, %) | 402 | 76 (37.8) | 73 (36.3) | 0.836 |
| Family history of SCD (n, %) | 402 | 31 (15.4) | 31 (15.4) | 1.000 |
| **Medical history** |  |  |  |  |
| Hypertension (n, %) | 402 | 48 (23.9) | 70 (34.9) | 0.021 |
| Diabetes (n, %) | 402 | 15 (7.5) | 10 (5.0) | 0.409 |
| Coronary artery disease (n, %) | 402 | 14 (7.0) | 11 (5.5) | 0.680 |
| **Imaging parameters** |  |  |  |  |
| LAD (mm) | 402 | 39.1±6.1 | 39.6±5.5 | 0.400 |
| MWT (mm) | 402 | 23.3±5.3 | 22.8±5.1 | 0.340 |
| LVEDD (mm) | 402 | 43.8±4.3 | 43.9±4.6 | 0.795 |
| LVEF (%) | 402 | 60.3±6.2 | 60.4±6.3 | 0.913 |
| LVOT gradient (mmHg) | 402 | 28 (6–87) | 16 (5–79) | 0.121 |
| †Among the study population, 258 patients underwent baseline 24-hour Holter monitoring. HCM, hypertrophic cardiomyopathy; ICD, implantable cardioverter-defibrillator; LAD, left atrial diameter; LVEDD, left ventricular (LV) end-diastolic diameter; LVEF, LV ejection fraction; LVOT, LV outflow tract; MWT, maximal wall thickness; NSVT, non-sustained ventricular tachycardia; NYHA, New York Heart Association; SCD, sudden cardiac death. | | | | |

| **Table S11. Indications for surgical myectomy among patients in the discovery cohort.** | |
| --- | --- |
| **Primary indications** | **Number of patients** |
| Obstructive HCM with severe symptoms (NYHA III–IV) despite maximally tolerated medical therapy | 92 |
| Obstructive HCM with severe chest pain attributable to LVOTO despite maximally tolerated medical therapy | 22 |
| Obstructive HCM with recurrent syncope/near-syncope attributable to LVOTO despite maximally tolerated medical therapy | 36 |
| Symptomatic obstructive HCM with anomalous papillary muscle requiring surgical intervention | 16 |
| Symptomatic obstructive HCM with primary mitral valve disease requiring surgical intervention | 26 |
| Symptomatic obstructive HCM with multivessel coronary artery disease requiring surgical intervention | 11 |
| Symptomatic obstructive HCM with severe aortic stenosis requiring surgical intervention | 2 |
| Obstructive HCM with NYHA II with left atrial enlargement with symptomatic atrial fibrillation | 11 |
| Symptomatic obstructive HCM with SAM-related moderate-to-severe mitral regurgitation despite medical therapy | 30 |
| Total | 246 |
| HCM, hypertrophic cardiomyopathy; LVOTO, left ventricular outflow tract obstruction; NYHA, New York Heart Association; SAM,systolic anterior motion. | |

| **Table S12. Detailed causes of cardiac death across study cohorts.** | | | | | | | |
| --- | --- | --- | --- | --- | --- | --- | --- |
| **Cohort** | **Cardiovascular Death** | | | | | | |
|  | **Total** | ***SCD** | | | **Heart failure-related death** | **Stroke-related death** | **Other cardiovascular death** |
|  |  | **SCD (actual death)** | **Sudden cardiac arrest** | **ICD shock** |  |  |  |
| **Discovery cohort** | **14** | **5 (35.7)** | **2**  **(14.3)** | **0 (0.0)** | **1 (7.1)** | **0 (0.0)** | **6 (42.9)** |
| Low *CECR1* | 14 | 5 (35.7) | 2  (14.3) | 0 (0.0) | 1 (7.1) | 0 (0.0) | 6 (42.9) |
| High *CECR1* | 0 | 0  (0.0) | 0  (0.0) | 0 (0.0) | 0 (0.0) | 0 (0.0) | 0 (0.0) |
| **Beijing validation cohort** | **53** | **24 (45.3)** | **3**  **(5.7)** | **1 (1.9)** | **16 (30.2)** | **4 (7.5)** | **5 (9.4)** |
| Low CECR1 | 46 | 24 (52.2) | 3  (6.5) | 1 (2.2) | 9 (19.6) | 4 (8.7) | 5 (10.9) |
| High CECR1 | 7 | 0 (0.0) | 0  (0.0) | 0 (0.0) | 7 (100.0) | 0 (0.0) | 0 (0.0) |
| **Xi'an validation cohort** | **16** | **7 (43.8)** | **1**  **(6.2)** | **2 (12.5)** | **2 (12.5)** | **1 (6.2)** | **3 (18.8)** |
| Low CECR1 | 14 | 6 (42.9) | 1  (7.1) | 1 (7.1) | 2 (14.3) | 1 (7.1) | 3 (21.5) |
| High CECR1 | 2 | 1 (50.0) | 0  (0.0) | 1 (50.0) | 0 (0.0) | 0 (0.0) | 0 (0.0) |
| *Patients resuscitated from sudden cardiac arrest and those received appropriate ICD shock therapy were also recorded as SCD. ICD, implantable cardioverter-defibrillator; SCD, sudden cardiac death. | | | | | | | |

| **Table S13. Detailed causes of cardiovascular death in discovery cohort.** | | |
| --- | --- | --- |
| **Case** | **Detailed Event Description** | **Event Classification** |
| Case1 | Postoperative death following Morrow septal myectomy | Other cardiovascular death |
| Case2 | The patient died of cardiac disease reported by family | Other cardiovascular death |
| Case3 | Death due to myocardial infarction | Other cardiovascular death |
| Case4 | Postoperative death following Morrow septal myectomy | Other cardiovascular death |
| Case5 | Sudden death while taking a bus on the day of hospital follow-up | Sudden cardiac death |
| Case6 | Cardiac arrest with successful resuscitation | Sudden cardiac arrest |
| Case7 | The patient had severe heart failure symptoms prior to death reported by family | Heart failure-related death |
| Case8 | Sudden unexpected death reported by family | Sudden cardiac death |
| Case9 | Sudden unexpected death reported by family | Sudden cardiac death |
| Case10 | Sudden loss of consciousness while climbing stairs, with restoration of spontaneous breathing after cardiopulmonary resuscitation, followed by 1 day of inpatient treatment | Sudden cardiac arrest |
| Case11 | Sudden unexpected death reported by family | Sudden cardiac death |
| Case12 | Sudden death during sport activities | Sudden cardiac death |
| Case13 | The patient died of cardiac disease reported by family | Other cardiovascular death |
| Case14 | The patient died of cardiac disease reported by family | Other cardiovascular death |
| ICD, implantable cardioverter-defibrillator. | | |

**Table S14. Detailed causes of cardiovascular death in the Beijing validation cohort.**

| **Case** | **Detailed Event Description** | **Event Classification** |
| --- | --- | --- |
| Case 1 | Death following sudden loss of consciousness | Sudden cardiac death |
| Case 2 | Cardiac arrest with successful resuscitation | Sudden cardiac arrest |
| Case 3 | Sudden unexpected death reported by family | Sudden cardiac death |
| Case 4 | Passed away during nocturnal sleep without prodromal symptoms | Sudden cardiac death |
| Case 5 | Sudden death during way to work | Sudden cardiac death |
| Case 6 | Sudden unexpected death reported by family | Sudden cardiac death |
| Case 7 | Sudden death while taking a bus on the day of hospital follow-up | Sudden cardiac death |
| Case 8 | Passed away during nocturnal sleep without prodromal symptoms | Sudden cardiac death |
| Case 9 | Cardiac arrest at an airport with successful resuscitation | Sudden cardiac arrest |
| Case 10 | Sudden cardiac death at home on day 4 post-discharge | Sudden cardiac death |
| Case 11 | The patient had severe heart failure symptoms prior to death reported by family | Heart failure-related death |
| Case 12 | The patient died of cardiomyopathy-induced heart failure reported by family | Heart failure-related death |
| Case 13 | Sudden unexpected death reported by family | Sudden cardiac death |
| Case 14 | The patient had severe heart failure symptoms prior to death reported by family | Heart failure-related death |
| Case 15 | Sudden unexpected death reported by family | Sudden cardiac death |
| Case 16 | In-hospital heart failure death | Heart failure-related death |
| Case 17 | In-hospital heart failure death | Heart failure-related death |
| Case 18 | Sudden unexpected death reported by family | Sudden cardiac death |
| Case 19 | Death due to ventricular fibrillation during sleep | Sudden cardiac death |
| Case 20 | In-hospital heart failure death | Heart failure-related death |
| Case 21 | The patient died of cardiac disease reported by family | Other cardiovascular death |
| Case 22 | Sudden unexpected death reported by family | Sudden cardiac death |
| Case 23 | Appropriate ICD therapy (shock) | ICD shock |
| Case 24 | Death due to ventricular fibrillation | Sudden cardiac death |
| Case 25 | Death due to myocardial infarction | Other cardiovascular death |
| Case 26 | Sudden collapse and death at work | Sudden cardiac death |
| Case 27 | Death due to cerebral infarction | Stroke-related death |
| Case 28 | Sudden unexpected death reported by family | Sudden cardiac death |
| Case 29 | Sudden unexpected death reported by family | Sudden cardiac death |
| Case 30 | Sudden unexpected death reported by family | Sudden cardiac death |
| Case 31 | Death due to intracerebral hemorrhage following cerebral infarction | Stroke-related death |
| Case 32 | Sudden unexpected death reported by family | Sudden cardiac death |
| Case 33 | Death due to cerebral infarction | Stroke-related death |
| Case 34 | Death due to myocardial infarction | Other cardiovascular death |
| Case 35 | Death due to intracerebral hemorrhage | Stroke-related death |
| Case 36 | The patient died of heart failure reported by family | Heart failure-related death |
| Case 37 | Sudden unexpected death, failed resuscitation during transport to the hospital | Sudden cardiac death |
| Case 38 | The patient died of heart failure reported by family | Heart failure-related death |
| Case 39 | Sudden unexpected death, failed resuscitation during transport to the hospital | Sudden cardiac death |
| Case 40 | In-hospital heart failure death | Heart failure-related death |
| Case 41 | Sudden loss of consciousness while climbing stairs, with restoration of spontaneous breathing after cardiopulmonary resuscitation, followed by 1 day of inpatient treatment | Sudden cardiac arrest |
| Case 42 | The patient died of heart failure reported by family | Heart failure-related death |
| Case 43 | The patient died of heart failure reported by family | Heart failure-related death |
| Case 44 | Death following unsuccessful heart transplantation | Heart failure-related death |
| Case 45 | Sudden unexpected death reported by family | Sudden cardiac death |
| Case 46 | In-hospital heart failure death | Heart failure-related death |
| Case 47 | Death due to myocardial infarction | Other cardiovascular death |
| Case 48 | Death due to myocardial infarction | Other cardiovascular death |
| Case 49 | Death due to ventricular fibrillation | Sudden cardiac death |
| Case 50 | Sudden unexpected death reported by family | Sudden cardiac death |
| Case 51 | The patient had severe heart failure symptoms prior to death reported by family | Heart failure-related death |
| Case 52 | The patient had severe heart failure symptoms prior to death reported by family | Heart failure-related death |
| Case 53 | The patient had severe heart failure symptoms prior to death reported by family | Heart failure-related death |
| ICD, implantable cardioverter-defibrillator. | | |

| **Table S15. Detailed causes of cardiovascular death in the Xi'an validation cohort.** | | |
| --- | --- | --- |
| **Case** | **Detailed Event Description** | **Event Classification** |
| Case 1 | Death due to myocardial infarction | Other cardiovascular death |
| Case 2 | Sudden unexpected death reported by family | Sudden cardiac death |
| Case 3 | Postoperative death following Morrow septal myectomy | Other cardiovascular death |
| Case 4 | Postoperative death following Morrow septal myectomy | Other cardiovascular death |
| Case 5 | Death due to ventricular fibrillation | Sudden cardiac death |
| Case 6 | Sudden unexpected death reported by family | Sudden cardiac death |
| Case 7 | Sudden unexpected death reported by family | Sudden cardiac death |
| Case 8 | Appropriate ICD therapy (shock) | ICD shock |
| Case 9 | Death due to cerebral infarction | Stroke-related death |
| Case 10 | Sudden unexpected death reported by family | Sudden cardiac death |
| Case 11 | Sudden unexpected death reported by family | Sudden cardiac death |
| Case 12 | Sudden unexpected death reported by family | Sudden cardiac death |
| Case 13 | In-hospital cardiac arrest, successfully resuscitated via defibrillation | Sudden cardiac arrest |
| Case 14 | Appropriate ICD therapy (shock) | ICD shock |
| Case 15 | The patient died of heart failure reported by family | Heart failure-related death |
| Case 16 | In-hospital heart failure death | Heart failure-related death |
| ICD, implantable cardioverter-defibrillator. | | |

**Table S16. Raw FPKM data.**

Please refer to the accompanying Excel file “Raw FPKM data”.

| **Table S17. List of antibodies used in this study.** | | |
| --- | --- | --- |
| **Name** | **Supplier** | **Cat no.** |
| Adenosine Deaminase 2/CECR1 Antibody | NOVUS | NBP1-89238 |
| GAPDH Monoclonal antibody | Proteintech | 60004-1-Ig |

| **Table S18. Primer sequences for real-time qPCR assay.** | | |
| --- | --- | --- |
| **Gene** | **Species** | **Sequence** |
| *CECR1* | Human | ACCATGACGAAGAGTGGTCAG |
|  |  | CCATTCGGATGGATTCTGCG |
| *GAPDH* | Human | AGATCCCTCCAAAATCAAGTGG |
|  |  | GGCAGAGATGATGACCCTTTT |

**STROBE Checklist of this study**

**Uncropped Western blot images**

**CECR1**

55kDa marker

70kDa marker

Cardiovascular Death

Survival

**GAPDH**

Survival

Cardiovascular Death

40kDa marker

35kDa marker
